# Divergent transcription is associated with promoters of transcriptional regulators

**DOI:** 10.1186/1471-2164-14-914

**Published:** 2013-12-23

**Authors:** Cyrille Lepoivre, Mohamed Belhocine, Aurélie Bergon, Aurélien Griffon, Miriam Yammine, Laurent Vanhille, Joaquin Zacarias-Cabeza, Marc-Antoine Garibal, Frederic Koch, Muhammad Ahmad Maqbool, Romain Fenouil, Beatrice Loriod, Hélène Holota, Marta Gut, Ivo Gut, Jean Imbert, Jean-Christophe Andrau, Denis Puthier, Salvatore Spicuglia

**Affiliations:** 1 Technological Advances for Genomics and Clinics (TAGC), Case 928, 163 Avenue de Luminy, 13288, Marseille cedex 09, France; 2 Aix-Marseille Université, UMR1090 TAGC, Marseille F-13288, France; 3 INSERM, UMR1090 TAGC, Marseille F-13288, France; 4 TGML, IBiSA Platform, Parc Scientifique de Luminy, Marseille, France; 5 Centre d’Immunologie de Marseille-Luminy, Campus de Luminy, Case 906, 13288 Marseille cedex 9, France; 6 CNRS UMR6102, Marseille, France; 7 Inserm, U631 Marseille, France; 8 Centre Nacional D’Anàlisi Genómica, Parc Científic de Barcelona, Baldiri i Reixac, 08028 Barcelona, Spain; 9 Polytech Marseille, Parc Scientifique de Luminy, Marseille, France; 10 Present address: CNRS, Aix-Marseille Université, IGS UMR7256, Marseille, France

**Keywords:** Divergent transcription, lncRNA, Bidirectional promoter, GC skew, Developmental transcription factor

## Abstract

**Background:**

Divergent transcription is a wide-spread phenomenon in mammals. For instance, short bidirectional transcripts are a hallmark of active promoters, while longer transcripts can be detected antisense from active genes in conditions where the RNA degradation machinery is inhibited. Moreover, many described long non-coding RNAs (lncRNAs) are transcribed antisense from coding gene promoters. However, the general significance of divergent lncRNA/mRNA gene pair transcription is still poorly understood. Here, we used strand-specific RNA-seq with high sequencing depth to thoroughly identify antisense transcripts from coding gene promoters in primary mouse tissues.

**Results:**

We found that a substantial fraction of coding-gene promoters sustain divergent transcription of long non-coding RNA (lncRNA)/mRNA gene pairs. Strikingly, upstream antisense transcription is significantly associated with genes related to transcriptional regulation and development. Their promoters share several characteristics with those of transcriptional developmental genes, including very large CpG islands, high degree of conservation and epigenetic regulation in ES cells. In-depth analysis revealed a unique GC skew profile at these promoter regions, while the associated coding genes were found to have large first exons, two genomic features that might enforce bidirectional transcription. Finally, genes associated with antisense transcription harbor specific H3K79me2 epigenetic marking and RNA polymerase II enrichment profiles linked to an intensified rate of early transcriptional elongation.

**Conclusions:**

We concluded that promoters of a class of transcription regulators are characterized by a specialized transcriptional control mechanism, which is directly coupled to relaxed bidirectional transcription.

## Background

Transcription of eukaryotic genomes generates a myriad of non-coding RNAs that show complex overlapping patterns of expression and regulation [1]. The complexity of the eukaryotic transcriptome, transcribed by RNA Polymerase (Pol) II, goes far beyond the coding genome and expands to many short RNA populations (such as miRNAs, siRNAs, piwiRNAs, eRNAs, TSS-RNAs) as well as long non-coding RNAs (lncRNAs) [2]. LncRNAs form a heterogeneous group of RNAs transcribed from intergenic or intragenic regions, which vary in length from 200 nucleotides to over 100 kb [3]. Intragenic non-coding transcripts might be further subdivided depending on the way they overlap protein-coding genes and/or the orientation with respect to protein-coding genes (sense or antisense) [4]. Although the biological relevance of many non-coding transcripts has been unambiguously established, this unanticipated level of complexity has led to the notion of pervasive transcription, which refers to the fact that transcription is not restricted to well-defined functional features, such as genes [5-7].

A large proportion of lncRNAs are transcribed in antisense orientation of protein-coding genes, with which they often share sequence complementarities [8,9]. Antisense RNAs could potentially exert a regulatory function on their corresponding sense mRNA at different levels. Recent findings have shown that some antisense transcripts act as epigenetic regulators of gene expression and chromatin remodeling [8], while others play a role at the level of translation efficiency [10]. Besides these transcripts, the existence of non-coding antisense transcripts emanating from the promoters of protein-coding genes (i.e. head-to-head conformation) has also emerged as a widespread phenomenon from yeast to mammals [11]. On the one hand, the presence of short bidirectional transcripts appears to be a hallmark of active promoters in mammals [12-14]. On the other hand, relatively longer non coding antisense transcripts can be detected upstream of most expressed genes in conditions where the RNA degradation machinery is inhibited [15-18]. Moreover, lncRNAs (including long intergenic non-coding RNAs or lincRNA) are preferentially localized at the vicinity of gene promoters in antisense orientation [4,19-21]. For instance, ~60% of lncRNAs expressed in ES cells were found to originate close to the TSS of protein-coding genes [21]. Whether long antisense transcripts emanating from bidirectional promoters have general functional implications in gene regulation is currently unknown [11].

In order to systematically identify and characterize bidirectional promoters associated with long non-coding antisense transcription, we took advantage of strand-specific RNA-seq experiments, which provide an unprecedented opportunity to analyze and categorize transcripts [22]. Thorough analyses of RNA-seq data from early developing thymocytes and other mouse tissues indicated that long-range bidirectional transcription is an intrinsic property of a class of promoters whose associated genes mainly encode for transcriptional regulators involved in development and cell differentiation. Accordingly, these promoters are characterized by large CpG islands, high degree of conservation and are generally repressed by Polycomb complexes in ES cells. Moreover, they display a unique GC skew profile, while the associated coding genes have large first exons, both properties likely reminiscent of their bidirectional activity. Surprisingly, coding genes associated with upstream antisense lncRNAs display an increased rate of immature transcription, highlighting an additional level of transcriptional control. Thus, expression of long non-coding antisense transcripts appears as a common feature of a subset of mammalian protein-coding gene promoters with functional implications for gene regulation.

## Results

### Systematic identification of genes associated with long upstream antisense transcripts

**Figure 1:**
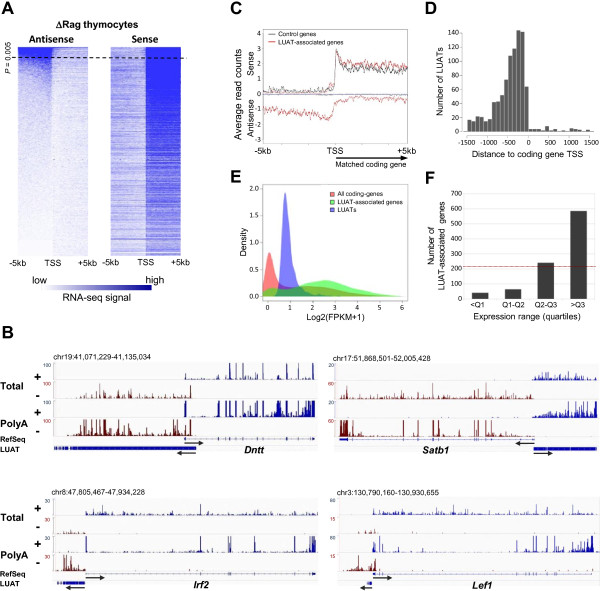
**Identification of genes associated with long upstream antisense transcripts in DN thymocytes. A)** Heatmap showing the Total RNA-seq signal from ΔRag (DN) thymocytes (SoliD platform) found in the 5 kb region surrounding the TSS of all non-overlapping Refseq genes. Signal was computed based on the number of reads per 100 bp binned regions originated from either the antisense or sense strand with respect to gene orientation (left and right panels, respectively). The heatmap is ordered according to the antisense signal for the [-5 kb; 0] region. The threshold for significantly expressed antisense transcripts is shown by a dotted line (see Methods section). **B)** Examples of genes associated with LUAT in ΔRag thymocytes. The Total and PolyA RNA-seq signals for the plus and minus strands are shown. Arrows indicate transcript orientation. The scales and genomic coordinates are shown on the left and top of each panel, respectively. Note that the scales were independently fixed for the plus and minus strands in order to properly visualize sense and antisense transcripts. **C)** Average profiles of PolyA- RNA-seq signal in ΔRag thymocytes for LUAT-associated genes (red line) and a control set of similarly expressed genes (black line). Signals corresponding to the orientation of the coding genes are represented as positive values while antisense signals as negative values. **D)** Histogram of the positions of 5′ end of LUATs relative to the TSS of their associated coding-genes. **E)** Distribution of expression of all coding genes (red), LUAT-associated genes (green) and LUATs (blue) in ΔRag thymocytes. **F)** Number of LUAT-associated genes in each expression quartile of all coding genes (Q1 = 3.05e-6 FPKM; Q2 = 0.013 FPKM; Q3 = 1.99 FPKM). The red line indicates the expected (random) distribution.

We sought to assess whether production of long antisense transcripts is a general feature of mammalian gene promoters. To this goal, we initially performed strand-specific paired-end Total RNA-seq with high sequencing depth from ΔRag thymocytes (Additional file 1: Table S1), corresponding mainly to CD4^-^CD8^-^ T-cell precursors (hereafter, double negative or DN thymocytes). We selected the set of protein-coding RefSeq transcripts whose promoter regions (from -5 kb to the transcription start site, TSS) do not overlap with transcripts of any other coding gene (a total of 17,186 transcripts; see Methods). We then calculated the total RNA-seq signal in the sense and antisense orientation for the region -5 kb to +5 kb with respect to each TSS and ordered the selection in function of the level of upstream antisense (AS) transcription (from -5 kb to the TSS; Figure 1A). Using a stringent threshold (*p* < 0.005; see Methods) we found 6.8% (1,177) of coding RefSeq transcripts to be associated with long upstream antisense transcripts (hereafter, LUATs), of which 236 overlap with previously annotated non-coding transcripts. Several examples are shown in Figure 1B. Interestingly, these antisense transcripts are generally polyadenylated as shown by the average profile of strand-specific and PolyA-enriched RNA-seq signal generated from ΔRag thymocytes (Figure 1B and C; Additional file 1: Table S1). To confirm our observation in a different tissue, we analyzed strand-specific Total RNA-seq data from mouse embryonic kidney [23] and obtained consistent results (Additional file 1: Table S1 and Additional file 2: Figure S1).

In order to infer the structure of LUATs, we used the transcript assembly tool Cufflinks [24,25]. We selected antisense Cufflinks transcripts starting within the region +/- 1.5 kb around the TSS and longer than 200 nt, and inferred antisense transcript models for 992 out of 1,177 RefSeq genes associated with divergent transcription in ΔRag thymocytes, as defined above (Additional file 3: Table S2). As expected, LUATs have very low or no coding potential as determined by PhyloCSF [26] analyses (Additional file 4: Figure S2). Assessment of subcellular localization of LUATs using recently published RNA-seq data obtained from fractionated chromatin-associated, nucleoplasmic and cytoplasmic transcripts (Bhatt et al. [27]), showed that they remains mainly associated with the chromatin fraction (Additional file 5: Figure S3), consistent with their lack of coding potential. Interestingly, 58% of antisense transcripts start within the region 500 bp upstream the TSS of the associated-coding genes (Figure 1D), suggesting that both sense and antisense transcripts originate from the same promoter elements.

LUATs were found to be expressed at relatively low level with a median expression value of 0.8 fragments per kilobase per million fragments mapped or FPKM (Figure 1E; see also Methods for details on quantification of LUAT expression). Strikingly however, the LUAT-associated coding genes are expressed at high levels (median expression value 3.9 FPKM; Figure 1E). Indeed, expression levels of the majority of genes displaying divergent promoters were found to be above the 3rd quartile of expression value distribution in ΔRag thymocytes (Figure 1F). Overall, these results suggest that antisense transcription is initiated from active coding-gene promoters, leading to concomitant expression of the two divergent transcripts.

### LUAT-associated genes are related to transcription and developmental functions

**Figure 2:**
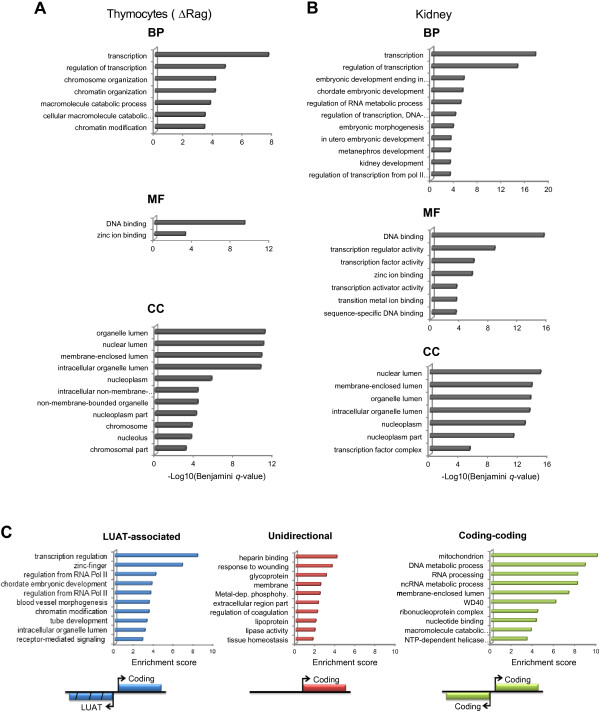
**Functional analysis of LUAT-associated genes. A-B)** Functional enrichment analyses for LUAT-associated genes found in ΔRag thymocytes **(A)** and embryonic kidney **(B)**. Significant GO terms for Molecular Function (MF), Biological Process (BP) and Cellular Component (CC) with a Benjamini-corrected *p* < 10^-3^ are shown. Note that using this threshold, a set of similarly expressed control genes retrieved no significant enrichment for GO terms. **C)** Enrichment scores of functional groups found using the Functional Classification Tool from DAVID [77]. Results are shown for LUAT-associated genes found in the multi-tissue analysis, bidirectional protein-coding gene pairs (coding-coding) and genes with unidirectional promoters. The top ten groups are shown for each set of genes. The functional groups are named based on the term with the lowest *p* value found in each group.

**Figure 3:**
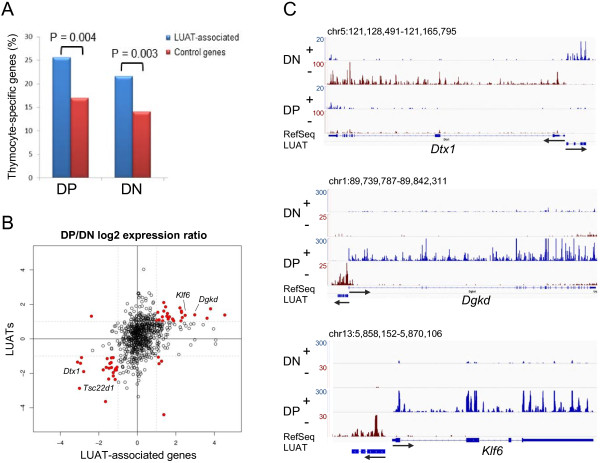
**Regulation of LUAT and associated genes during early T-cell differentiation. A)** Percentage of thymocyte-specific genes in the LUAT-associated gene sets from either DP or DN (ΔRag) thymocytes or in control sets of similarly expressed genes (see Methods). The *p* values results from a Chi-2 test are shown. **B)** Scatterplot showing the log2 ratio of Total RNA-seq signals (FPKM) between DN (ΔRag) and DP thymocytes (both from Illumina platform) for LUAT and associated genes. Shown in red are LUATs and associated gene pairs which are both considered as differentially expressed (2-fold change). **C)** Examples of co-regulated LUATs and associated genes between DN (ΔRag) and DP thymocytes. The Total RNA-seq signal for the plus and minus strands is shown. Legends are as in Figure 1B.

The above results indicated that a substantial fraction of mammalian promoters sustain divergent transcription of lncRNA/mRNA gene pairs. We next assessed whether LUAT-associated genes were enriched for specific categories of genes. We found that this set of genes is highly enriched with transcription- and chromatin regulation-related GO terms (Figure 2A). This observation was specific to LUAT-associated genes as compared to a control set composed of genes with similar expression level distribution, but without antisense transcription, which was not significantly enriched for any GO terms (considering Benjamini-corrected *p* < 0.001 as a threshold). Strikingly, the list of LUAT-associated genes includes most transcription regulators known to be important for early T-cell differentiation [28], including *Tcf7* (TCF1), *Lef1*, *Tcf12* (E47), *Satb1, Dntt, Gfi1*, *Myb*, *Tox*, *Notch1, Bcl11a*, *Rorc* (Rorγt) and *Ikzf1*. Consistent with a tissue-specific function, LUATs are significantly associated with a higher proportion of thymocyte-specific genes (Figure 3A) and with genes involved in T cell differentiation (ToppGene analysis for “Mouse Phenotypes” [29]; Bonferroni-corrected *p*-value: 0.004), as compared to the control set. Enrichment for transcription- and development-related functions was also found with LUAT-associated genes isolated from kidney RNA-seq data (Figure 2B; Additional file 2: Figure S1), comprising important regulators of kidney development, such as *Irx2*, *Irx3*, *Hnf1b*, *Lhx1* and *Smad4*.

To obtain a more general view of the functional relevance of LUAT-associated genes, we analyzed recently published directional and PolyA-enriched RNA-seq data from 17 mouse tissues [30]. Although the sequencing depth was generally low, we were able to isolate LUATs for all analyzed tissues and to annotate a non-redundant set of 1,118 LUAT-associated genes (Additional file 6: Table S3). Consistent with the initial studies from thymus and kidney (Figure 2A and B), this set of genes was highly enriched for genes involved in transcription regulation and developmental functions (Figure 2C). In contrast, head-to-head coding genes (coding-coding) or randomly selected genes with unidirectional promoters are not enriched for transcription-related functions (Figure 2C; Note that control sets were chosen by selecting genes for which the breadth of expression matched those of the LUAT-associated gene set; Additional file 7: Table S4). Next, we addressed the question of whether LUAT-associated genes were specifically enriched for developmental genes involved in transcription regulation. We found that LUAT-associated genes, but not control genes, were enriched in the set of genes annotated for both “Developmental process” (GO:0032502, biological process) and “Transcription factor activity” (GO:0003700, molecular function) GO terms (*p* < 1 × 10^-8^, Fisher’s exact test; see Material and Methods). In conclusion, LUAT-associated genes are commonly involved in transcription regulation related to developmental functions.

### LUAT and their associated coding-genes are co-regulated throughout cell differentiation and development

The co-expression of the two divergent transcripts suggests that the expression of LUATs and their neighboring genes might be co-regulated throughout development and cell differentiation. To address this hypothesis, we first compared two subsequent stages of early T-cell development. During normal T-cell differentiation, preTCR-induced signaling leads DN thymocytes to cross the so-called β-selection checkpoint, which results in massive cell proliferation and the induction of a developmental process marked by the expression of both CD4 and CD8 co-receptors, thus generating DP thymocytes [31]. To determine whether LUATs and associated genes were co-regulated during the β-selection process, we used previously published Total RNA-seq from DP thymocytes [32] and produced a new set of RNA-seq data from ΔRag (DN) thymocytes, using the same RNA-seq procedure and sequencing platform (Additional file 1: Table S1). We then selected a non-redundant list of 758 LUAT expressed in either DN or DP cell stages and compared their differential expression ratio along with the expression ratio of the associated coding-genes (Figure 3B; Additional file 8: Table S5). Interestingly, we observed a significant association between developmental regulation of LUAT and their associated genes when considering transcripts with an expression ratio of at least twofold (*p* < 0.0001; Chi-squared test). Examples of co-regulated LUAT-gene pairs are shown in Figure 3C.

To have a more thorough dynamical view of the regulated expression of LUATs and their associated genes, we analyzed recently published RNA-seq data from several stages of early T-cell differentiation [33]. Although the absence of strand-specific information did not allow genome-wide isolation of LUAT in these data sets, visual inspection of the RNA-seq revealed clear examples where the LUAT and the associated gene followed the same kinetics throughout T-cell differentiation (Additional file 9: Figure S4). In the same line, we also observed a tight co-regulation of LUAT and associated gene pairs between thymocytes and embryonic kidney (Additional file 2: Figure S1B).

**Figure 4:**
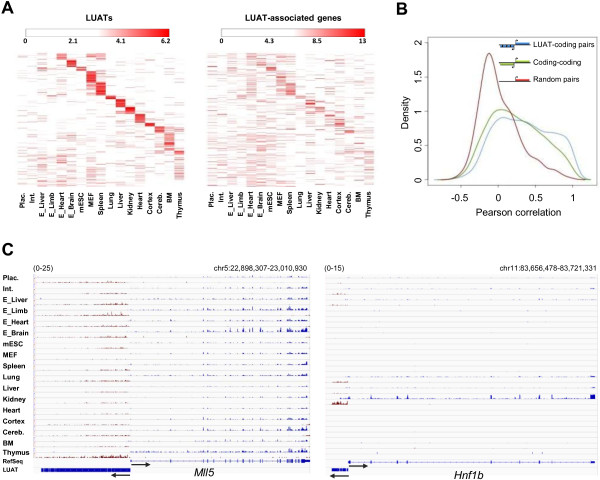
**Co-expression of LUAT and associated genes. A)** Left panel: heatmap of expression profiles of LUATs in the 17 indicated tissues. Expression profiles were partitioned using K-means algorithm (k = 14). Right panel: heatmap of expression profiles of LUAT-associated genes in the same tissues. Lines are ordered according to the corresponding LUATs. **B)** Distribution of Pearson correlation coefficient between expression values of indicated gene pairs across the 17 tissues. Red: randomly paired genes (with unidirectional promoters); green: head-to-head protein-coding genes; blue: LUAT and their associated genes. **C)** Examples of LUAT and associated genes across the 17 tissues. The PolyA RNA-seq signals for the plus and minus strands are shown in blue and red, respectively. The scale is indicated in the top-left of each panel.

One expectation from this observation is that the expression of LUATs and their associated genes would be correlated across different tissues. To address this possibility, we analyzed the expression patterns of the 1,118 LUATs and their associated genes found in the multi-tissue analysis. The vast majority of LUATs exhibit tissue-specific expression patterns as underlined by unsupervised clustering of expression profiles (k-means algorithm; Figure 4A) and the restricted number of tissues where each of them was found (Additional file 10: Figure S5). Moreover, these LUATs and their coding neighbors are more correlated to each other than random gene pairs, and even slightly more than head-to-head protein-coding gene pairs (Figure 4B). In agreement, we found many examples where LUATs expression is strictly associated with the expression of their neighboring genes (Figure 4C). Taken together, these results suggest that LUAT expression likely reflects the activity of associated coding-gene promoters throughout cell differentiation and development.

### LUAT-associated promoters share characteristics with those of developmentally regulated genes

**Figure 5:**
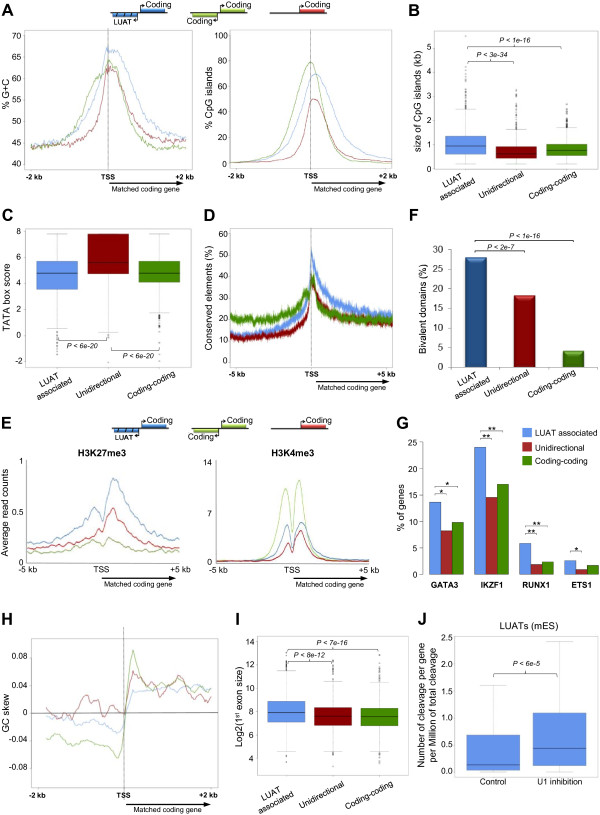
**Characterization of sequence content and regulatory features of LUAT-associated promoters. ** Results in A-F and H-I are shown for the three set of genes described in Figure 2C. **A)** Average GC content (left panel) and percentage of CpG islands (right panel) around the TSS (bidirectional promoters are centered on the TSS from the genes that has been used to match the expression with the LUAT associated genes). **B)** Boxplot showing the distribution of sizes of the CpG islands overlapping the 2 kb region around the TSS (when several CpG islands were found, the sum was calculated). **C)** Boxplot showing the distribution of TATA box motif scores found in a 500 bp region around the TSS. **D)** Percentage of sequences with a conserved element at each position around the TSS. **E)** Average profiles of indicated ChIP-seq data in ES cells around the TSS. **F)** Percentage of genes having a bivalent domain in their promoter, as defined in [37]. Statistical significances were computed using the hypergeometric test. **G)** Percentage of genes associated with lymphoid-specific transcription factors. The histogram shows the overlap between indicated transcription factor peaks and regions around TSS (+/-5 kb) for the genes selected in DP thymocytes. Statistical significances were computed using the hypergeometric test (**p value < 0.01; *p value < 0.05). **H)** Average GC skew profiles, computed as (#G-#C)/(#G+#C). I) Boxplot showing the distribution of first exon length. **J)** The normalized number of cleavage sites in antisense orientation identified in two control and two U1 inhibition experiments in ES cells [43] was computed for a 5 kb region upstream the TSS of genes for which an associated LUAT was expressed in mouse ES cells (FPKM >1). In panels **B**, **C**, **I** and **J**, *p* values of the Wilcoxon rank sum test are shown.

The close proximity between LUAT and TSS of associated coding genes, along with the tight correlation of their expression, strongly suggest sharing of common regulatory elements. Hence, we asked whether the bidirectional property of LUAT-associated promoters might be linked to intrinsic sequence specificities. We compared several sequence characteristics between the promoters of the three gene sets described above. We found that GC content differs between these sets. In the region upstream of the TSS, both LUAT-associated and coding-coding gene promoters have a significantly higher GC content than unidirectional promoters (*t*-test in region [-500 bp; TSS]; *p* < 10^-50^ and *p* < 10^-81^, respectively), whereas in the downstream region, LUAT-associated promoters have a higher GC content than the unidirectional and coding-coding gene sets (*t*-test in region [TSS; +500 bp], *p* < 10^-38^ and *p* < 10^-45^, respectively) (Figure 5A, left panel). Moreover, 80% of LUAT-associated and 89% of coding-coding gene promoters are covered by CpG islands within the region [-500 bp; +500 bp], as compared to only 56% of unidirectional gene promoters (Figure 5A, right panel). Strikingly however, analysis of CpG island size demonstrated that LUAT-associated gene promoters contain particularly longer CpG islands (Figure 5B; 46.2% of LUAT-associated promoters have a CpG island size greater than 1 kb, as compared to 23.6% and 26.5% of bidirectional coding-gene and unidirectional promoters, respectively). Sequence motif analyses revealed that both LUAT-associated and coding-coding gene promoters are depleted of TATA boxes, as compared to unidirectional promoters (Figure 5C). Finally, we found that LUAT-associated promoters contain more conserved elements than the other two sets in regions close to and downstream of the TSS (*t*-test in region [TSS; +500 bp]; *p* < 10^-166^ and *p* < 10^-224^, respectively; Figure 5D). Interestingly, it has been described that developmentally regulated genes are associated with Genomic Regulatory Blocks (GRB) which are highly conserved genomic regions characterized by a number of unique features, including very large CpG islands and TATA-box depletion [34,35]. Therefore, the bidirectional property of LUAT-associated promoters might be linked to intrinsic regulatory properties related to genes encoding for transcriptional and developmental regulators.

Developmental genes have also been shown to be actively repressed by Polycomb complexes in Embryonic Stem (ES) cells, and therefore are often found associated with trimethylation of H3 lysine 27 (H3K27me3) [36]. By analyzing ChIP-seq data from ES cells [37], we found that LUAT-associated promoters were specifically enriched for H3K27me3 within the 2 kb around the TSS, as compared to unidirectional and coding-coding gene sets (Mann–Whitney U test: *p* = 0.007 and *p* = 0.001, respectively), but not for H3K4me3 (Figure 5E). Moreover, they were more frequently associated with bivalent domains (Figure 5F), composed of concomitant H3K27me3 and H3K4me3 peaks, a feature related to silencing of developmental genes in ES cells, while keeping them poised for activation [37,38]. One additional expectation for developmentally regulated promoters is that they should be bound by tissue specific transcription factors. To test this, we analyzed ChIP-seq data performed in DP thymocytes for a series of lymphoid-specific transcription factors, including GATA3 [39], Ikaros [40], ETS1 [32] and RUNX1 (this study). These transcription factors were found in a higher frequency at LUAT-associated promoters active in DP thymocytes, as compared with a set of control genes (Figure 5G). Thus, LUAT-associated promoters appear to be regulated in a more specific way than other similarly expressed genes.

Overall, we found that LUAT-associated promoters share characteristics with those of developmentally regulated genes. It seems likely that the divergent transcription observed at LUAT-associated promoters is linked to intrinsic genomic characteristics of these promoters.

### Bidirectional transcription at LUAT-associated promoters is linked to a specific GC skew profile and longer first exon

Directionality of transcription is thought to be mediated, at least in part, by an asymmetric distribution of G and C content between the two DNA strands around the promoter, a property known as GC skew [41], possibly constraining the orientation of the transcription initiation complex. GC-rich promoters are characterized by a significant excess of G over C residues (positive GC skew) immediately downstream the TSSs [42]. To test the link between GC skew and bidirectionality, we computed GC skew profiles for each of the three gene promoter groups described above. As expected, unidirectional genes show a positive GC skew immediately downstream the TSS, while coding-coding genes show two sharp and inverted GC skew peaks, one negative and one positive, respectively upstream and downstream the TSS (Figure 5G). Strikingly, LUAT-associated genes also display two inverted GC skew profiles, but the GC bias is less pronounced than at head-to-head coding genes at both sides of the TSS (*p* < 5 × 10^-5^ and *p* < 2 × 10^-10^; *t*-test for the regions [-500 bp; TSS] and [TSS; +500 bp], respectively). In addition, the positive GC skew downstream the TSS is also less pronounced than at unidirectional genes (*p* < 1 × 10^-5^; *t*-test for the region [TSS; +500 bp]). This result suggests that bidirectional transcription at LUAT-associated promoters might be linked to a unique GC skew profile resulting in lower constraints on the directionality of the transcription initiation complex.

Promoter-proximal 5′ splicing sites and first exon length have been recently suggested to play a role in directionality of transcription [43-45]. We computed the average length of the first exon of genes in each gene set (Figure 5I). Strikingly, the set of LUAT-associated genes has the longest first exon with a median length of 242 bp, compared to 195 bp and 190 bp for the unidirectional and coding-coding gene sets. Consistently, 5′ splicing sites are relatively depleted immediately downstream the TSS of LUAT-associated genes, as compared to the control set of genes (Additional file 11: Figure S6). To assess whether splicing might play a role in controlling the expression of LUATs, we analyzed recent published data of 3′ ends of polyadenylated RNA-seq in mouse ES cells in which U1 small nuclear ribonucleoprotein (snRNP) has been functionally inhibited [43]. Interestingly, the expression (as measured by the level of 3′ ends of polyadenylated RNA) of a selection of LUAT normally expressed in ES cells (FPKM > 1) is significantly increased after inhibition of U1 snRNP (Figure 5J), as compared to control ES cells. Taken together, these observations indicate that a unique GC skew profile along with large first exon might both contribute to the bidirectionality of transcription at LUAT-associated genes.

### LUAT-associated genes harbor specific chromatin features

**Figure 6:**
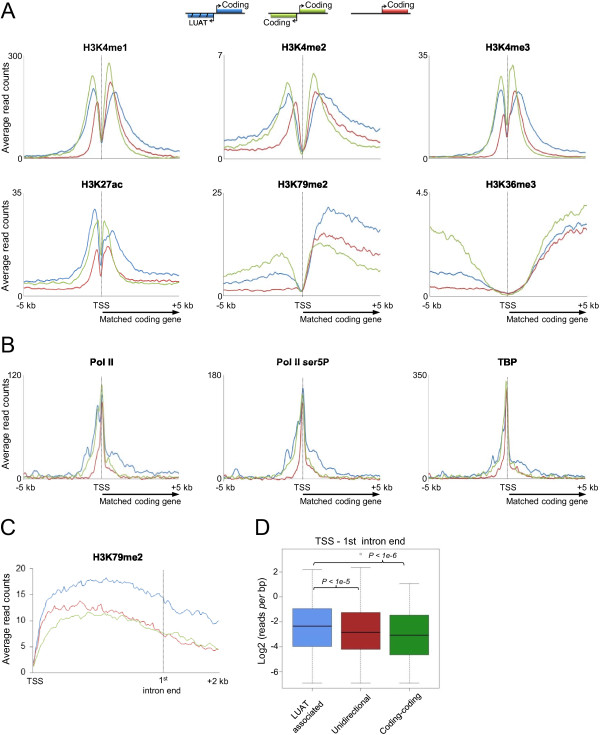
**Chromatin characteristics of LUAT-associated promoters in DP thymocytes.** Average profiles of ChIP-seq signals of the indicated histone modifications **(A)** and general transcription factors **(B)**. The 5 kb region around the TSS of the genes included in the indicated sets are shown: LUAT-associated genes, bidirectional coding-gene (coding-coding) and unidirectional genes. The three gene sets were selected ensuring a similar distribution of gene expression levels in DP thymocytes (see Methods). **C)** Average profile of ChIP-seq signals for H3K79me2 in rescaled region ranging from the TSS to the end of the first intron. **D)**  Boxplots showing the distribution of reads per bp for H3K79me2 within the region comprising the TSS and the end of the first intron (*p*-values of the Wilcoxon rank sum test are shown).

In order to assess whether LUAT-associated promoters display specific chromatin features, we analyzed several histone modification marks and general transcription factors in DP thymocytes that were either performed in this study or already published [32] (Figure 6). We compared the surrounding regions of three sets of promoters displaying similar expression level distribution based on Total RNA-seq signal at exons (FPKM) in DP thymocytes: LUATs-associated promoters, unidirectional promoters, as well as promoters of bidirectional coding gene pairs (coding-coding set). As expected, we found chromatin features common to both sets of bidirectional promoters (Figure 6A). Bidirectional promoters display higher and/or wider level of histone modifications linked to open and active chromatin (H3K4me1/2/3 and H3K27ac) at the region immediately upstream of the TSS (Figure 6A), consistent with the bidirectional activity at these promoters. This property is also associated with additional peaks upstream of the TSS for either total or initiating (Ser5 phosphorylated: Ser5P) Pol II, as well as TBP (Figure 6). Furthermore, significant levels of histone modifications linked to early (H3K79me2) or late (H3K36me3) transcription elongation were observed in the region upstream of bidirectional promoters, confirming the fact that these regions undergo productive transcription (Figure 6A).

Strikingly, we also found evidences for chromatin features specific to LUAT-associated promoters. Although the three gene sets are similarly enriched for H3K36me3 within the coding gene body, the LUAT-associated genes display higher levels of H3K79me2 (Figure 6A). H3K79me2 is generally enriched at the 5′ end of expressed genes comprising the first exon and intron and mark the transition between early (immature) and late (productive) transcription elongation [46]. To have a more precise view of the differential enrichment in H3K79me2, we compared the H3K79me2 profiles within rescaled regions comprising the TSS to the end of 1^st^ intron (Figure 6C and D). Indeed, we observed that H3K79me2 remains significantly higher throughout the first intron of LUAT-associated genes as compared to the control gene sets. As the genes from the three sets express similar amounts of productive transcription (as assessed by both Total and PolyA RNA-seq counting at exons; Figure 6A), our results most likely suggest an actual increased rate of early (immature) transcription elongation from LUAT-associated promoters.

In agreement with an increased rate of early transcriptional elongation at LUAT-associated genes, we observed aspecific enrichment of Pol II (either total or Ser5P) and TBP within the 5′ region of LUAT-associated gene-bodies (Figure 6B and Additional file 12: Figure S7), indicating extended accumulation of the initiating and/or early elongating transcription complex [47]. To further investigate the possibility of a transcriptional pause immediately downstream the TSS, we analyzed the average profiles in DP thymocytes of additional general transcription factors (GTFs), including TAF1, TFIIB, TFIIE and TFIIH (Additional file 13: Figure S8). In all cases, we observed a significant enrichment of the GTFs downstream the TSS. Finally, consistently with the transcriptional pause being directly linked to divergent transcription, we also observed a significant and specific enrichment of (Ser5P)Pol II and GTFs around a region 1 kb upstream the TSS of LUAT associated genes (Additional file 12: Figure S7). Of particular interest is the overall enrichment in TFIIH complex around the TSS of LUAT-associated genes, which play a key role in transcription initiation by phosphorylating Pol II at Ser5 [47]. Thus, our results indicate increased Pol II pausing at both sides of LUAT-associated promoters.

### Accumulation of immature transcripts at LUAT-associated genes

**Figure 7:**
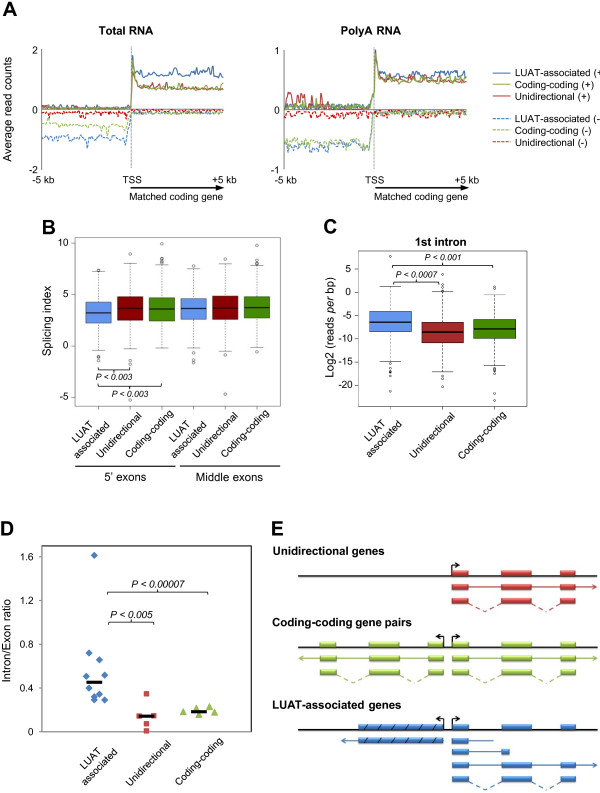
**LUAT-associated promoters are prone to pervasive transcription. A)** Average profiles of Total and PolyA RNA-seq signals in DP thymocytes, for the three set of similarly expressed genes. Signals coming from plus and minus strands are indicated by solid and dashed lines, respectively. **B)** Splicing index calculated for the 5′ and middle exons for the three set of similarly expressed genes in DP thymocytes. **C)** Boxplots showing the density of Total RNA-seq reads *per* bp in the same orientation as the matched coding genes and within the first intron of the three group of genes in DP thymocytes. Statistical significance was assessed by the Mann–Whitney U test. **D)** Intron/exon ratio of individual genes for the three gene sets in DP thymocytes assessed by reverse transcription quantitative PCR. Relative transcript levels at the first intron and the last exon of each gene was estimated based on a standard dilution of genomic DNA. Statistical significance were assessed using Wilcoxon rank sum tests. **E)** Schematic representation of RNA processing at the three different classes of gene loci. Exons are shown by rectangles (or stripped rectangles in the case of LUATs). Solid and dotted lines represent immature (unspliced) and processed (spliced) transcripts, respectively. Our results suggest that LUAT-associated genes display an increased rate of immature transcripts.

Early elongation and H3K79me2 enrichment are generally associated with the 5′ intronic sequences and splicing events [46]. Thus, to further explore the hypothesis of an accumulation of immature transcripts at LUAT-associated genes, we compared the average profiles around the TSS of Total and PolyA RNA-seq levels for the three set of equally expressed genes (Note that these sets of genes have equal distribution of exonic FPKM based on either Total or PolyA RNA-seq, data not shown). We found that Total-RNA signal downstream of the TSS is higher for LUAT-associated genes, while PolyA-RNA signal is similar among the three gene sets (Figure 7A, compare left and right panels). While the PolyA RNA-seq signals result only from complete (fully processed) transcripts, Total RNA-seq signals result from both immature (partial or unprocessed) and complete transcripts. Thus, a relative enrichment of Total RNA-seq, as compared to PolyA RNA-seq signal, is indicative of either increased rate of immature transcription or expression of less stable transcripts. However, the observed results could not be attributed to differences in transcript stability as the three gene sets display equivalent levels of H3K36me3, which is generally coupled to productive elongation (Figure 6A).

To directly assess whether LUAT-associated genes produce increased level of immature transcripts (i.e. more unspliced transcripts), we computed the splicing index across the three gene sets. As shown in Figure 7B, the splicing index is significantly reduced between the first two exons of LUAT-associated genes as compared to the other set of genes, while no differences are observed at the middle exons. This suggests an increased rate of immature transcription at the 5′ region of LUAT-associated genes. Consistently, we found that first introns of LUAT-associated genes display higher density of Total RNA-seq signal as compared to the control gene sets (Figure 7C), though there is no significant differences in first intron length between the three set of genes (data not shown). Significant enrichment of immature transcripts within the first intron of LUAT-associated genes was also confirmed by RT-qPCR analysis of individual genes (Figure 7D). All in all, LUAT-associated gene promoters are clearly more prone to induce immature transcription than other bidirectional or unidirectional gene promoters, indicating a less restricted control of Pol II pausing around the TSS, likely leading to divergent and pervasive transcription.

## Discussion

Here, we used directional RNA-seq from primary mouse tissues to directly and systematically characterize antisense transcripts. We have found that a significant fraction of gene promoters sustains expression of long non-coding antisense transcripts (here named LUATs). The LUAT/coding gene pairs are usually co-regulated throughout cell differentiation and development and generally function as transcriptional regulators. LUAT-associated promoters share several characteristics of promoters of developmentally regulated genes, including large CpG islands, high degree of conservation and epigenetic regulation during development. Moreover, the bidirectional transcription at these promoters appears to be linked to a specific GC skew profile and particularly longer first exons. Finally, LUAT-associated promoters display distinctive epigenetic features reflecting an intensified rate of early transcriptional elongation. Overall, our results support the view that promoters of a subclass of transcriptional regulators are characterized by a specialized mechanism of Pol II transcription, in which Pol II recruitment is directly coupled to relaxed bidirectional transcription.

Previous studies have shown that lincRNAs are preferentially located near protein-coding genes in divergent orientation and that their expression is often correlated [19,20,48]. However, the stringent criteria generally used to define lincRNAs (e.g., the presence of at least two exons) likely impaired a comprehensive identification of lncRNA transcripts, including those in divergent orientation from coding genes. A recent study has indeed described the abundance of divergently transcribed LncRNA/mRNA gene pairs in ES cells [21]. Although this and the present works likely described the same kind of antisense transcripts, our study largely complement and extend the previous study by using a more direct approach to identify upstream antisense lncRNAs solely based on the RNA-seq signal and by demonstrating their existence in many primary tissues and throughout T cell differentiation. Moreover, we show here that divergent transcription is clearly associated with a subset of genes coding for transcriptional regulators, and we propose a functional link between divergent transcription and gene expression regulation.

As suggested previously the presence of short bidirectional transcripts appears to be a hallmark of active promoters in mammals [12,13], generally associated with paused Pol II around the TSS. It has also been previously described that expression of upstream unstable transcripts (also called PROMPTs) are a common characteristic of Pol II transcribed genes [16,17]. Although some overlaps might exist between PROMPTs and LUATs, LUATs differ from exosome-sensitive PROMPTs transcripts. Firstly, LUATs are detected at significant levels without inhibition of the RNA exosome degradation machinery. Secondly, they are substantially longer than PROMPTs. Thirdly, they are associated with a specific category of genes. Hence, while many coding gene promoters, essentially those containing CpG islands [49], have the intrinsic property of bidirectional transcription [11], production of long antisense transcript is generally inhibited or are rapidly degraded at most loci.

### Divergent transcription is linked to intrinsic sequence properties shared with developmental gene promoters

We have found that LUAT-associated genes frequently encode TFs involved in cell differentiation and development. This is consistent with previous studies in mouse, human and zebra fish suggesting that large gene deserts flanking TF genes, with roles in embryonic development, preferentially harbor lincRNAs [50-54]. As such, these genes are expected to be subjected to fine tune regulation. Accordingly, we found that their promoters appears to be bound by lineage specific TFs (Figure 5G). Another striking characteristic of LUAT-associated promoters is the presence of very large CpG islands (Figure 5A and B), a feature shared with developmental gene promoters [34], but not observed at bidirectional coding-gene promoters. As the promoters of these genes are enriched for CpG rich regions and are prone to pervasive transcription, it is expected that dedicated repressive mechanisms might be in place to control their expression. In line with this, we have found that LUAT-associated genes are specifically enriched for H3K27me3 (Figure 5E) and for bivalent domains (Figure 5F) in ES cells, thus suggesting Polycomb-mediated regulation of these genes. As suggested elsewhere [55], large CpG islands (as those found at LUAT-associated promoters) might represent a favored recruitment platform for Polycomb-associated complexes and thus play an important role in transcriptional regulation of key developmental genes. Whether large CpG islands are required for divergent transcription from LUAT-associated promoters will deserve further investigation.

### A link between antisense and immature transcription?

Our results indicate an unexpected link between divergent lncRNA/mRNA transcription and premature termination of coding transcripts. Indeed, LUAT-associated genes are characterized by a significant accumulation of immature transcripts within the 5′ region of the genes (Figure 7). Our results most likely suggest that intrinsic properties of LUAT-associated promoters induce a specialized mechanism of Pol II transcription, in which recruitment of the enzyme is directly coupled to pervasive bidirectional transcription. Several arguments point to this direction. The presence of a TATA box is generally linked to strong directionality of transcription. Consistent with their bidirectional feature, LUAT-associated promoters are essentially depleted of TATA box (Figure 5C). Although the absence of a TATA box might be required for bidirectional transcription, as has been suggested for coding-coding promoters [56,57], it might not be sufficient as some TATA-less promoters still show strong directionality [57]. In addition, we have also observed that LUAT-associated promoters display two inverted, though moderated, GC skew profiles around the TSS (Figure 5E). It has been shown that a positive GC skew immediately upstream the TSS favors the formation of so-called R-loop structures [42], which are long, stable and three stranded RNA/DNA structure formed during transcription [58]. It has also been previously suggested that positive GC skew immediately after the TSS, and subsequent R loop formation, may serve to correct the lack of directionality in the initial steps of transcription [42]. This correction might be mediated by the ability of R loops to elicit transcriptional pausing [59-61]. Finally, 5′ splice sites and first exons have been recently shown to play a role in promoter directionality [43-45]. It is suggested that U1 snRNP binding at 5′ splice sites might help to stabilize Pol II recruitment at the promoter and enforce sense transcription. Moreover, the length of first exon appears to influence Pol II stability at promoters and transcription accuracy [45]. Indeed, genes with long first exon have Pol Il accumulation downstream the TSS and increased proportion of antisense transcripts [45], reminiscent of what we have observed at LUAT-associated loci. Strikingly, functional inhibition of U1 snRNP in ES cells resulted in accumulation of premature cleavage of coding gene transcripts [43], but also increased expression of antisense transcripts (Figure 5J). Consequently, spatial separation of promoter and 5′ splice sites might directly impact both directionality and transcription accuracy. It is plausible that, at LUAT-associated genes, 5′ splice sites are too distant from the TSS to ensure proper directionality of transcription, thus favoring bidirectional transcription. Taking all into account, we favor a hypothesis whereby both immature and bidirectional transcription at a specialized class of gene promoters are directly linked to intrinsic sequence properties, including TATA box depletion, unique GC skew profile and long first exon.

### Functional relevance of bidirectional transcription

The specific association of antisense transcripts with genes related to transcription regulation and development strongly suggests that divergent transcription might be directly or indirectly involved in the tight regulation of these genes. In line with a direct role of LUATs, several examples have demonstrated a functional regulation by mammalian antisense transcripts in *cis*[11,62,63]. This regulation might act at multiple levels, from modifying local chromatin to enabling regional signal spreading [11,62] or controlling translation efficiency [10], yet in the latter case an overlapping stretch with coding exons is required. Alternatively, divergent transcription might reflect an intrinsic property of promoters of genes coding for developmental regulators. The finding that H3K79me2, a mark of early elongation, was found higher at LUAT-associated genes (Figure 6A) suggests the level of early elongating transcription is increased at these genes. This was further supported by a relative higher ratio of Total *versus* PolyA levels within the first intron, as well as decreased splicing efficiency (Figure 7A-C). Thus, our findings clearly indicate that LUAT-associated promoters are more prone to pervasive and/or immature transcription (Figure 7D).

What can be the functional relevance of this pervasive transcription? If divergent transcription and non-productive gene expression are linked, this will imply that the initiation step of transcription is less controlled at these promoters, likely leading to pervasive transcription in both directions. In this line, an appealing hypothesis will be that expression of LUAT-associated genes is regulated also at the level of transcript maturation and/or elongation efficiency. This might reflect a checkpoint for coupling elongation and RNA processing, as previously suggested [64]. Although some genes are regulated by Pol II pausing in close proximity to the TSS [65], others are reported to be efficiently transcribed into precursor transcripts, while the efficiency of transcript processing is being regulated [66,67]. Generally, only a portion of the Pol II that assemble at the promoter enters into early elongation [68]. This entry is characterized by Pol II Ser5 phosphorylation and chromatin modifications that are specific to transcription initiation and early elongation (such as H3K79me2). Before transcribing further, the polymerase passes a 5′ checkpoint where it pauses, terminates, or commits to productive elongation. If the polymerase does not proceed through this checkpoint, transcription will be terminated producing an unstable transcript. If the polymerase proceeds through the checkpoint, it will enter into productive elongation that is associated with characteristic chromatin modifications (such as H3K36me3) and Pol II Ser2 phosphorylation [47]. In this context, rapid transcriptional induction might be facilitated by the active release of polymerase molecules that have initiated transcription, but are paused downstream the TSS. Thus, pausing during early elongation may provide both a kinetic ‘window of opportunity’, as well as an interaction surface, to facilitate additional levels of regulation of the nascent RNA before the transition to productive elongation.

Another related and not exclusive hypothesis would be that bidirectional promoter activity increase the stochasticity of gene expression, as suggested for antisense expression in yeast [69]. It is well known that expression of developmental regulators, including key transcription factors, is often regulated stochastically during cell differentiation, influencing cell and developmental decisions [70,71]. It is therefore plausible that LUAT expression might reflect a dedicated mechanism to induce stochastic expression of developmental regulators by modulating the rate of pervasive (i.e. non-productive) transcription. In any case, our observations might shed light on a new mechanism representing the outcome of an evolutionary pressure to control the expression of a subclass of genes coding for transcriptional regulators.

## Conclusions

We have found that divergent transcription of lncRNA/mRNA gene pairs is significantly associated with genes related to transcriptional regulation and development. Their promoters share several characteristics with those of developmental transcription factors, display a unique GC skew profile and are associated with genes harboring long first exons, reminiscent of their bidirectional activity. Unexpectedly, we also found that the 5′ region of the associated coding genes harbor a specific accumulation of H3K79me2 epigenetic mark, as well as initiating Pol II complexes, both of which are linked to an intensified rate of early transcriptional elongation. These results strongly suggest a functional link between divergent transcription and the regulation of genes coding for developmental transcription factors.

Altogether, our findings which indicate that, at a subset of transcription factor gene promoters, divergent and pervasive transcription are linked, might illustrate an additional mechanism for regulating the regulators, in a rather sophisticated system for fine-tuning mammalian gene expression.

## Methods

### Mice and cell preparations

Homozygous *Rag2*-deficient (ΔRag) mice [72] were housed under specific pathogen free conditions and handled in accordance with French and European directives. All mice were bred on a C57Bl/6 J background and were killed for analysis between 4 and 6 weeks of age. DN and DP thymocytes were purified as previously described [32,73].

### RNA extraction and library preparation

Total RNA from 10×10^6^ thymocytes of ΔRag mice was extracted as previously described [32]. Strand-specific preparation, sequencing and processing of Total and PolyA RNA samples were carry out as previously described [32]. RNA quantity and quality were verified using RNA Pico chips on a 2100 Bioanalyzer (Agilent).

### RNA-seq datasets

Paired-end stranded RNA-seq was performed with ribo-depleted Total RNA obtained from ΔRag thymocytes using SOLiD platform version 4. Single-end stranded RNA-seq was also performed on ribo-depleted Total and PolyA RNA obtained from ΔRag thymocytes using Illumina GAII sequencer. Strand-specific Total and Poly(A) RNA-seq from double-positive T-cells were obtained from SRA ftp site (SRX063934, SRX063935 respectively). RNA-seq data from thymocytes across development (DN1, DN2a, DN2b, DN3 and DP) where obtained from SRA ftp site (SRP007822). RNA-seq data performed using fractionated chromatin-, nucleoplasmic-, and cytoplasmic- associated transcripts where obtained from SRA ftp site (SRX100837, SRX100832 and SRX100827 respectively). RNA-seq data from mouse tissues were obtained from SRA (SRP006787). For quality filtering, sequencing read obtained from Illumina and SoliD platforms were quality trimmed using sickle (-q10) or csfasta_quality_filter (-m 8 -l 25 -s 14 -n 2), respectively. Selected reads (minimum length 25 nt) were then aligned to mm9 genome using TopHat (version 2.0.4) [24]. Gene annotations (gtf file) and indexes (nucleotide or color space) were obtained from TopHat website (mm9 iGenome). Multireads were rejected from all experiments. Additional information about mapping results for RNA-seq experiments is available in Additional file 1: Table S1.

### Quantitative RT-PCR

RNA was isolated from 6 weeks old C57BLK6 mice thymi using TRIzol^®^ reagent (Invitrogen). RNA quality was monitored with RNA Nano-6000 Chips and 2100- BioAnalyzer (Agilent). Two-step quantitative reverse transcription PCR (RT-qPCR) was performed using the Stratagene Mx3000P Sequence Detection System. Random hexamers and the reverse transcriptase SuperScript II (Invitrogen) were used for RNA reverse transcription. Quantitative PCR was performed with Syber^®^ Green PCR Mix (Applied Biosystem). Primers were designed in the first Intron and the last exon of selected unidirectional, coding-coding and LUAT-associated genes (primer sequences are provided in Additional file 14: Table S6). Relative transcript levels were estimated based on a standard dilution of genomic DNA and the intron/exon ratio was calculated for each gene.

### Identification of LUATs

We first selected the +/-5 kb regions around the TSS of all coding transcripts available from RefSeq database (mm9, UCSC). In case several TSSs originating from several isoforms of the same gene were distant from less than 100 bp, only one representative transcript was selected. To exclude coding gene whose promoter overlap any other coding transcript, we then filtered out transcripts whose upstream 5 kb region overlapped with any coding transcript from another gene both on positive and negative strand. Coverage was then measured (both on positive and negative strand) in binned regions (100 bp) around the TSS of the selected transcripts using coverageBed program (bedtools suite, version 2.13.3) [74] and expressed as log2(coverage +1). Transcripts were sorted according to the sum of bin coverage on the opposite strand of upstream 5 kb region. The subsequently obtained heatmap was visualized using treeview. In order to define a set of candidate coding genes displaying a significant signal in opposite strand within the upstream 5 kb regions, the same procedure (binning and coverage analysis) was applied to a set of 10,000 randomly selected intergenic regions. The distribution of the bin coverage sum obtained for all randomly chosen regions was used to define a threshold with *p*-value < 0.005. Cufflinks [24] was subsequently used to discover new isoforms and transcripts (using known transcript list as guide), and to perform assembly.

### Quantification of LUAT expression

We observed that inferred cufflinks transcripts appeared generally fragmented compared to the underlying RNA-seq signal, probably due to mapability issues (i.e. low complexity sequences), low expression levels or uneven coverage. Thus, to maximize the assembly of full length transcripts, cufflinks fragments closer than 800 bp were combined. We thus developed a python script that used novel Cufflinks transcripts located within 1.5 kb from the TSS of selected candidate coding genes as seed and extend them in 5′ and 3′ orientation while any novel cufflinks of length above 200 bp, present on the same strand and distant from less than 800 bp is found. A gtf file was subsequently produced containing coordinates of novel transcripts overlapping promoter regions. This file was merged with a gtf file containing coding genes and used for transcript abundance estimation (FPKM) using cufflinks (using the -G parameter). The list of LUAT/gene pairs, including genomic coordinates and FPKM, for the different data sets described in this study is provided in the Additional file 3: Table S2, Additional file 6: Table S3 and Additional file 8: Table S5.

### Definition of gene sets

Given the list of LUAT-associated genes found in DN or DP thymocytes, we first filtered out the genes having several alternative TSSs. We then generated two control sets of the same size. A set of coding genes with no overlapping transcript in their 5 kb upstream region (defined as unidirectional genes). A set of coding genes having another coding gene in their upstream region, oriented in the opposite direction, and with TSS separated less than 1.5 kb from each other (defined as coding-coding genes). The selection of the two control sets was then performed with a random sampling procedure implemented in R, ensuring similar distribution of gene expression (as measured by the exonic FPKM from Total RNA-seq data) in all 3 gene sets. In the case of coding-coding genes, the TSS used to anchor the average plots was the one corresponding to the matched expression. For multi-tissue analysis, where no unique reference expression level exists, the two control sets were chosen by selecting genes for which the maximum of expression across the 17 tissues matched those of the LUAT-associated gene list (Additional file 7: Table S4).

### Thymocytes-specific genes

We retrieved gene expression data from the GNF Gene Atlas [75], using samples from a large variety of tissues. For each gene, we computed a score of tissue-specificity TS_g,t_ = (e_g,t_ – Q3_g_)/(Q3_g_ – Q1_g_), where e_g,t_ is the expression of gene g in tissue t, Q1_g_ and Q3_g_ are the first and third quartiles in the distribution of expression values for gene g across all tissues. Genes with scores higher than 1 were considered as outliers of that distribution, so a gene g was called specific of a tissue t if TS_g,t_ > 1. For Figure 3A, we used scores associated to the thymus.

### ChIP-seq data and analysis

Chromatin preparation and immunoprecipation for ChIP was performed as described previously (Koch et al. [32]). H3K79me2 ChIP was performed from sonicated chromatin from 5 million DP thymocytes using 2 μg of antibody (ab3594, Abcam). Runx1 ChIP was performed from sonicated chromatin from 10 million DP thymocytes using 10 μg of antibody (ab3594, Abcam). ChIP samples were subsequently sequenced in either Genome Analyzer II (Illumina, USA; H3K79me2) or AB SOLiD V4.0 (Life Technologies; Runx1) according to the manufacturer’s instructions. ChIP-seq data from mouse DP thymocytes for Pol II, TBP, General transcription factors TAF1, TFIIB, TFIIE and TFIIH, as well as other histone modifications have been previously published (Koch et al. [32]) and were analyzed as described in (Koch et al. [32]). ChIP-seq datasets for additional transcription factors in DP thymocytes were downloaded from Gene Expression Omnibus (IKZF1: GSE32311, ETS1: GSE29362, GATA3: GSE20898 merged with GSE31233, Input: GSE31233 and GSE32311). H3K4me3 and H3K27me3 ChIP-seq data from mouse ES cells were obtained from [37]. To generate average profiles, mm9 Refseq genes annotations were used to extract values from wiggle files associated with selected regions. The selected regions are defined in a region of 5 kb before and after TSSs of gene list selections. A bin scores from wiggle files were used to interpolate around the TSS and generates the average profiles. For assessing binding of lymphoid-specific transcription factors in Figure 5G, peak calling was performed by using the Hypergeometric Optimization of Motif EnRichment (HOMER) tool (v4.1) [76] with default settings (FDR: 0.001; local and input fold enrichment: 4.0). We computed the overlap between transcription factors peaks and regions around TSS (+/- 5 kb) for the indicated group of genes.

### Functional enrichment analysis

GO term analysis were performed with DAVID [77]. In the analyses shown in Figures 2A and B, we selected, for each category, the terms with a Benjamini-corrected *p* value below 0,001 using the “Functional annotation chart” and default options. For the analyses shown in Figure 2C, we used “Functional annotation clustering” and selected the top 10 clusters retrieved for each gene set. Assessment for functional enrichment of “developmental transcription factor” in the list of LUAT-associated coding genes was performed using R and TBrowser database [78]. GO data were first retrieved and genes associated both to terms “developmental process” (GO term GO:0032502, biological process ontology) and “sequence-specific DNA binding transcription factor activity” (GO term GO :0003700, molecular function ontology) were defined as “developmental transcription factor”. A contingency table was then created using the list of genes annotated in both biological process and molecular function ontology as a reference. *p*-value was obtained using Fisher's exact test.

### Splicing index

For computation of splicing index, only coding RefSeq transcripts with at least 4 exons and FPKM above 0.1 were selected. The coverage of their exonic and intronic features was computed using coverageBed (from the bedtools suite) and a pseudo-count added to ensure non-zero values. FPKM values where then computed for each feature. The 5′ exonic signal was computed by averaging FPKM values corresponding to the first and second exons. For gene displaying an even number of exons the middle exonic RPKM was computed as the average signal between the two central exons whereas for genes displaying an odd number of exons the signal corresponding to the central exon was used. The splicing index corresponds to the log ratio between exonic FPKM value and intronic FPKM value.

### Availability of supporting data

Original ChIP-seq and RNA-seq data used in this study have been submitted to the NCBI Gene Expression Omnibus (GEO) (http://www.ncbi.nlm.nih.gov/geo/) under accession number GSE44578.

## Competing interests

The authors declare that they have no competing interests.

## Authors’ contributions

CL, MB, AB and DP performed the main bioinformatics analyses of the manuscript. AB, DP and RF processed deep sequencing data. AG analyzed ChIP-seq data from transcription factors. MY performed RT-qPCR assays, LV, JZC, FK, MAB performed RNA-seq and ChIP-seq experiments. BL, HL, JI, MG and IG performed deep sequencing. JCA, DP and SS conceived the study, and participated in its design and coordination. CL, DP and SS wrote the manuscript. All authors read and approved the final manuscript.

## Supplementary Material

Additional file 1: Table S1 Information about RNA-Seq datasets used in this study. The number of input reads and subsequent alignements are indicated. Click here for file

Additional file 2: Figure S1 Identification of genes associated with long upstream antisense transcripts in embryonic kidney. A) Heatmap showing the Total RNA-seq signal from mouse embryonic Kidney (Thiagarajan et al. [23]) found in a [-5000;+5000] region around the TSS of all non-overlapping Refseq genes. Signal was computed based on number of reads per 100 bp binned regions originated from either antisense or sense strand with respect to gene annotation (left and right panels, respectively). The heatmap is ordered according to the antisense signal for the [-5000;0] region. B) Example of genes associated with LUAT in mouse kidney. Total RNA-seq signal from embryonic kidney (Thiagarajan et al. [23]) and ΔRag DN thymocytes (SOLiD platform, this study) are shown. Signals from plus and minus strands are displayed in blue and red respectively. The *Dntt* gene is shown as an example of T-cell specific LUAT-associated gene. The arrow highlights the presence of a LUAT. Click here for file

Additional file 3: Table S2 LUAT and associated coding genes found in mouse DN thymocytes (SOLiD platform). Click here for file

Additional file 4: Figure S2 Assessment of coding potential. PhyloCSF assessement of LUAT coding potential. The Galaxy web server (https://main.g2.bx.psu.edu/) was used to extract MAF blocks from 46-way multiZ alignments using cufflink transcript coordinates as input. Corresponding genomic sequences for human (hg19), Mus musculus (mm9), Rattus norvegicus (Rn4), Bos taurus (bosTau4) and Canis familiaris (canFam2) were retrieved for each block. Blocks shorter than 50 bp (95% of mouse exons) or missing one of the selected species were discarded. In order to create a positive control list, a set of blocks with same length distribution was randomly selected in exons from coding transcripts. The PhyloCSF program was used to assess coding potential of both sets. The resulting log-likelihood ratios are reported in units of decibans. Click here for file

Additional file 5: Figure S3 LUATs are mainly found in the chromatin-associated fraction. To gain insight into the cellular localization of LUAT transcripts we analyzed recently published RNA-Seq data obtained from fractionated chromatin-associated, nucleoplasmic, and cytoplasmic transcripts from mouse macrophages (Bhatt et al. [27]; GEO serie: GSE32916). We performed assembly of divergent transcripts observed in these fractions (see “Identification of LUATs” in Methods section). A) boxplot displaying expression level as log2(FPKM + 1) for coding genes (blue) and LUATs (red) in the three different subcellular fractions. B) Representative examples of RNA-seq profiles from the three different subcellular fractions. Signal is provided for both plus and minus strands. The arrow highlights the presence of a LUAT. Click here for file

Additional file 6: Table S3 LUAT and associated coding genes found in the multi-tissue analysis. Click here for file

Additional file 7: Table S4 Coding-coding and unidirectional gene sets for which the maximum of expression across the 17 tissues matched those of LUAT-associated gene list. Click here for file

Additional file 8: Table S5 LUAT and associated coding genes found in mouse DN and DP thymocytes (Illumina platforme). Click here for file

Additional file 9: Figure S4 Dynamic regulation of LUAT and their associated genes through early T-cell differentiation. In order to define expression profiles of LUAT and associated coding-genes through discrete stages of thymocyte development we retrieved unstranded PolyA RNA-seq from GEO web site (GSE31234, Zhang at al. 2012). Unstranded RNA-seq signal is shown for DN1, DN2a, DN2b, DN3 and DP (black track). Signal obtained from ΔRag DN thymocytes (SOLiD platform, this study) is also shown to highlight the expected signals from the plus (blue) and minus (red) strands. The arrow highlights the presence of a LUAT. Click here for file

Additional file 10: Figure S5 Tissue-specificity of LUATs. The histogram shows the number of tissues in which a given LUAT was found in the multi-tissue analysis. Click here for file

Additional file 11: Figure S6 5′ splice site distribution. Analysis for 5′ splice site motifs (Jaspar database; ID SD0001.1) in the 500 nt regions downstream of TSS for the three group of genes used for the multi-tissue analyses (A) or LUATs (B). The *y* axis shows the cumulative fraction of regions having at least one predicted site after traversal of a given number of nucleotides, as indicated on the *x* axis. Click here for file

Additional file 12: Figure S7 Detailed view of TSS-centered ChIP-seq profiles for Pol II and TBP in DP thymocytes. Legends are as in Figure 6. The highlighted regions in pink correspond to the 500 nt regions analyzed in B. B) Number of reads within the indicated regions for the corresponding ChIP-seq experiments shown in A. The *p-*values of the Wilcoxon test are shown. Click here for file

Additional file 13: Figure S8 Detailed view of TSS-centered ChIP-seq profiles for the indicated general transcription factors in DP thymocytes. Legends are as in Figure 6. The highlighted regions in pink correspond to the 500 nt regions analyzed in B. B) Number of reads within the indicated regions for the corresponding ChIP-seq experiments shown in A. The *p-*values of the Wilcoxon test are shown. Click here for file

Additional file 14: Table S6 Primers used for the RT-qPCR assays. Click here for file
