# Supplementary material for: Divergent transcription is associated with promoters of transcriptional regulators
Source: BMC Genomics. 2013 Dec 23;14:914. doi: 10.1186/1471-2164-14-914 (PMC3882496; doi:10.1186/1471-2164-14-914)
Supplement: Additional file 1: Table S1 — Information about RNA-Seq datasets used in this study. The number of input reads and subsequent alignements are indicated. [file 1471-2164-14-914-S1.doc]

**Table S1:** Information about RNA-Seq datasets used in this study. The number of input reads and subsequent alignements are indicated.

| **Sample/tissue** | **RNA fraction** | **Platform** | **Sequencing characteristics** | **# of spots** | **# of Alignments** | **SRA_ID** | **Source** |
| --- | --- | --- | --- | --- | --- | --- | --- |
| ΔRag thymocytes | Total (ribo-depleted) | SOLiD 4 | Strand-specific; paired-end (50bp-35bp) | 523,138,985 | 238,621,400 | SRX233553 | This study |
| ΔRag thymocytes | Total (ribo-depleted) | Illumina GAII | Strand-specific; single-end (36bp) | 132,675,120 | 63,490,956 | SRX244276 | This study |
| ΔRag thymocytes | PolyA-enriched | Illumina GAII | Strand-specific; single-end (36bp) | 107,221,563 | 71,277,285 | SRX244277 | This study |
| DP thymocytes | Total (ribo-depleted) | Illumina GAII | Strand-specific; single-end (36bp) | 157,379,633 | 95,354,076 | SRX063934 | Koch et al. 2011 |
| DP thymocytes | PolyA-enriched | Illumina GAII | Strand-specific; single-end (36bp) | 129,154,580 | 101,210,955 | SRX063935 | Koch et al. 2011 |
| Macrophages | Chromatin fraction (ribo-depleted) | Illumina HiSeq 2000 | Strand-specific; single-end(50bp) | 83,908,878 | 56,451,773 | SRX100837 | Bhatt et al., 2012 |
| Kidney | Total (ribo-depleted) | SOLiD 4 | Strand-specific; single-end(50bp) | 416,727,040 | 60,325,274 | SRX033361 | Thiagarajan et al., 2011 |
| Macrophages | nucleoplasmic fraction (ribo-depleted) | Illumina HiSeq 2000 | Strand-specific; single-end(50bp) | 100,885,546 | 48,106,358 | SRX100832 | Bhatt et al., 2012 |
| Macrophages | Cytoplasmic fraction (ribo-depleted) | Illumina HiSeq 2000 | Strand-specific; single-end(50bp) | 81,242,654 | 26,134,877 | SRX100827 | Bhatt et al., 2012 |
| Fetal liver-derived DN1 | PolyA-enriched | Illumina GAII | Unstranded; single-end (38 bp) | 27,890,656 | 17,927,666 | SRX091684 | Zhang et al., 2012 |
| Fetal liver-derived DN1 | PolyA-enriched | Illumina GAII | Unstranded; single-end (38 bp) | 19,259,022 | 14,953,246 | SRX091685 | Zhang et al., 2012 |
| Fetal liver-derived DN2a | PolyA-enriched | Illumina GAII | Unstranded; single-end (38 bp) | 23,958,216 | 17,963,891 | SRX091686 | Zhang et al., 2012 |
| Fetal liver-derived DN2a | PolyA-enriched | Illumina GAII | Unstranded; single-end (38 bp) | 33,816,742 | 23,480,563 | SRX091687 | Zhang et al., 2012 |
| Fetal liver-derived DN2b | PolyA-enriched | Illumina GAII | Unstranded; single-end (38 bp) | 18,538,216 | 12,688,985 | SRX091688 | Zhang et al., 2012 |
| Fetal liver-derived DN2b | PolyA-enriched | Illumina GAII | Unstranded; single-end (38 bp) | 18,496,961 | 14,444,768 | SRX091689 | Zhang et al., 2012 |
| Fetal liver-derived DN2b | PolyA-enriched | Illumina GAII | Unstranded; single-end (38 bp) | 18,575,391 | 14,892,099 | SRX091690 | Zhang et al., 2012 |
| Thymic DN3 | PolyA-enriched | Illumina GAII | Unstranded; single-end (38 bp) | 18,689,345 | 12,592,481 | SRX091691 | Zhang et al., 2012 |
| Thymic DN3 | PolyA-enriched | Illumina GAII | Unstranded; single-end (38 bp) | 23,376,516 | 17,772,042 | SRX091692 | Zhang et al., 2012 |
| Thymic DP | PolyA-enriched | Illumina GAII | Unstranded; single-end (38 bp) | 18,894,429 | 10,316,186 | SRX091693 | Zhang et al., 2012 |
| Thymic DP | PolyA-enriched | Illumina GAII | Unstranded; single-end (38 bp) | 17,638,690 | 14,353,562 | SRX091694 | Zhang et al., 2012 |
| Bone marrow | PolyA-enriched | Illumina GAII | Strand-specific; single-end (30bp) | 27,199,485 | 15,624,033 | SRX062997 | Shen et al., 2012 |
| Cerebellum | PolyA-enriched | Illumina GAII | Strand-specific; single-end (30bp) | 69,113,217 | 38,806,416 | SRX062998 | Shen et al., 2012 |
| Cortex | PolyA-enriched | Illumina GAII | Strand-specific; single-end (30bp) | 65,243,500 | 40,947,270 | SRX062999 | Shen et al., 2012 |
| Heart | PolyA-enriched | Illumina GAII | Strand-specific; single-end (30bp) | 47,567,137 | 33,766,530 | SRX063000 | Shen et al., 2012 |
| Kidney | PolyA-enriched | Illumina GAII | Strand-specific; single-end (30bp) | 67,100,297 | 41,830,492 | SRX063001 | Shen et al., 2012 |
| Liver | PolyA-enriched | Illumina GAI | Strand-specific; single-end (30bp) | 49,033,021 | 30,298,341 | SRX063002 | Shen et al., 2012 |
| Lung | PolyA-enriched | Illumina GAII | Strand-specific; single-end (30bp) | 49,202,819 | 36,231,494 | SRX063003 | Shen et al., 2012 |
| Spleen | PolyA-enriched | Illumina GAII | Strand-specific; single-end (30bp) | 46,119,764 | 32,377,024 | SRX063004 | Shen et al., 2012 |
| MEF | PolyA-enriched | Illumina GAII | Strand-specific; single-end (30bp) | 67,635,087 | 42,342,985 | SRX063005 | Shen et al., 2012 |
| mESC | PolyA-enriched | Illumina GAII | Strand-specific; single-end (30bp) | 53,947,437 | 33,308,680 | SRX063006 | Shen et al., 2012 |
| E14.5-brain | PolyA-enriched | Illumina HiSeq 2000 | Strand-specific; fragment (36bp) | 40,216,147 | 10,209,854 | SRX113069 | Shen et al., 2012 |
| E14.5-heart | PolyA-enriched | Illumina HiSeq 2000 | Strand-specific; fragment (36bp) | 54,048,507 | 13,163,276 | SRX113070 | Shen et al., 2012 |
| E14.5-limb | PolyA-enriched | Illumina HiSeq 2000 | Strand-specific; fragment (36bp) | 39,774,740 | 8,949,898 | SRX113071 | Shen et al., 2012 |
| E14.5-liver | PolyA-enriched | Illumina HiSeq 2000 | Strand-specific; fragment (36bp) | 32,734,865 | 7,577,788 | SRX113072 | Shen et al., 2012 |
| Intestine | PolyA-enriched | Illumina HiSeq 2000 | Strand-specific; fragment (36bp) | 55,189,724 | 23,867,484 | SRX113073 | Shen et al., 2012 |
| Placenta | PolyA-enriched | Illumina HiSeq 2000 | Strand-specific; fragment (36bp) | 43,881,812 | 11,987,796 | SRX113075 | Shen et al., 2012 |
| Thymus | PolyA-enriched | Illumina HiSeq 2000 | Strand-specific; fragment (36bp) | 17,674,452 | 2,201,130 | SRX113077 | Shen et al., 2012 |
