# Supplementary figures and images for: Divergent transcription is associated with promoters of transcriptional regulators
Source: BMC Genomics. 2013 Dec 23;14:914. doi: 10.1186/1471-2164-14-914 (PMC3882496; doi:10.1186/1471-2164-14-914)

**A**

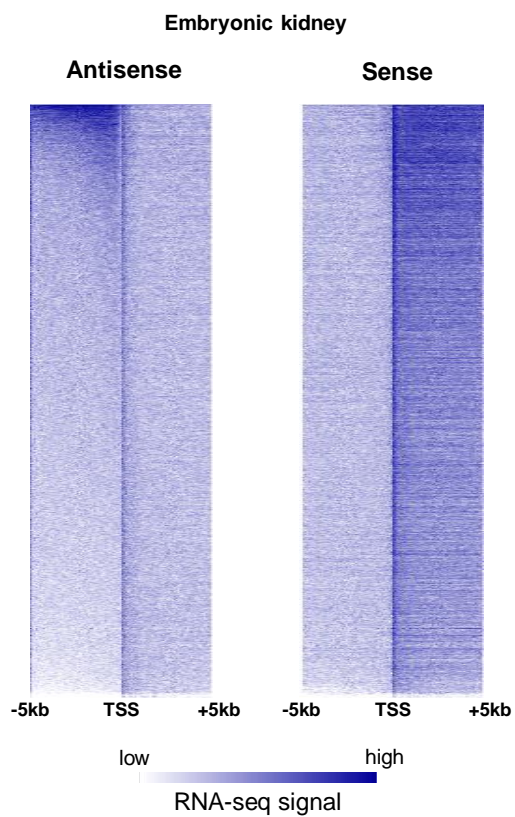

**B**

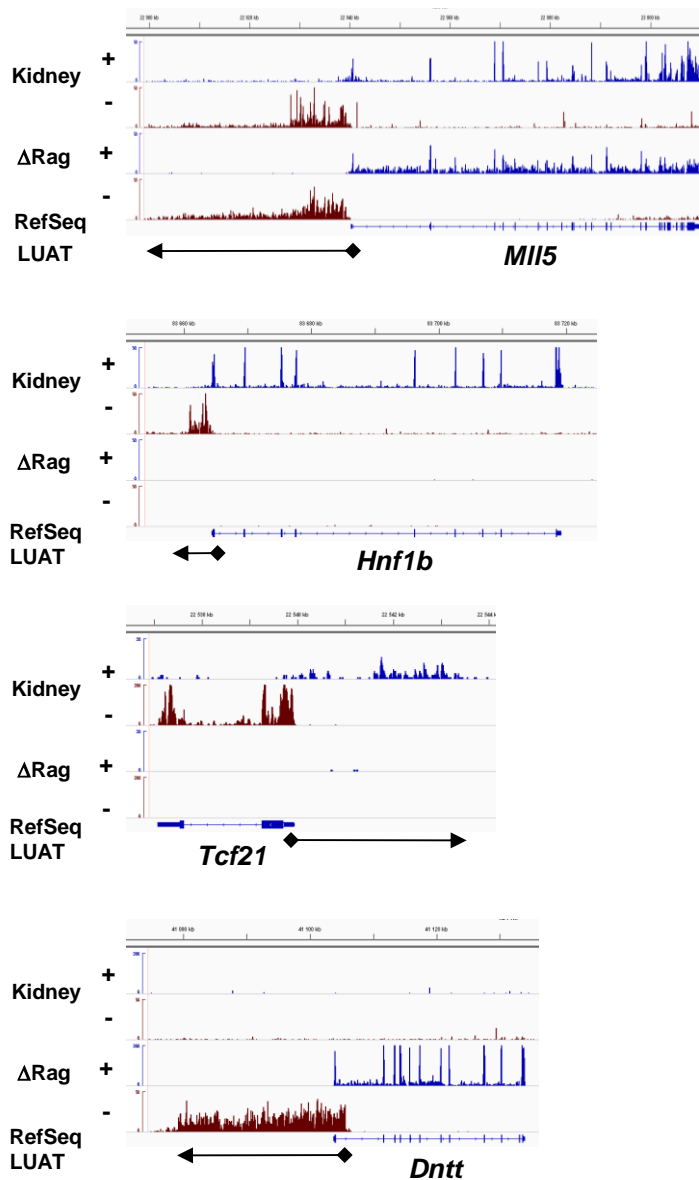

Supplement: Additional file 2: Figure S1 — Identification of genes associated with long upstream antisense transcripts in embryonic kidney. A) Heatmap showing the Total RNA-seq signal from mouse embryonic Kidney (Thiagarajan et al. [23]) found in a [-5000;+5000] region around the TSS of all non-overlapping Refseq genes. Signal was computed based on number of reads per 100 bp binned regions originated from either antisense or sense strand with respect to gene annotation (left and right panels, respectively). The heatmap is ordered according to the antisense signal for the [-5000;0] region. B) Example of genes associated with LUAT in mouse kidney. Total RNA-seq signal from embryonic kidney (Thiagarajan et al. [23]) and ΔRag DN thymocytes (SOLiD platform, this study) are shown. Signals from plus and minus strands are displayed in blue and red respectively. The Dntt gene is shown as an example of T-cell specific LUAT-associated gene. The arrow highlights the presence of a LUAT. [file 1471-2164-14-914-S2.pdf]

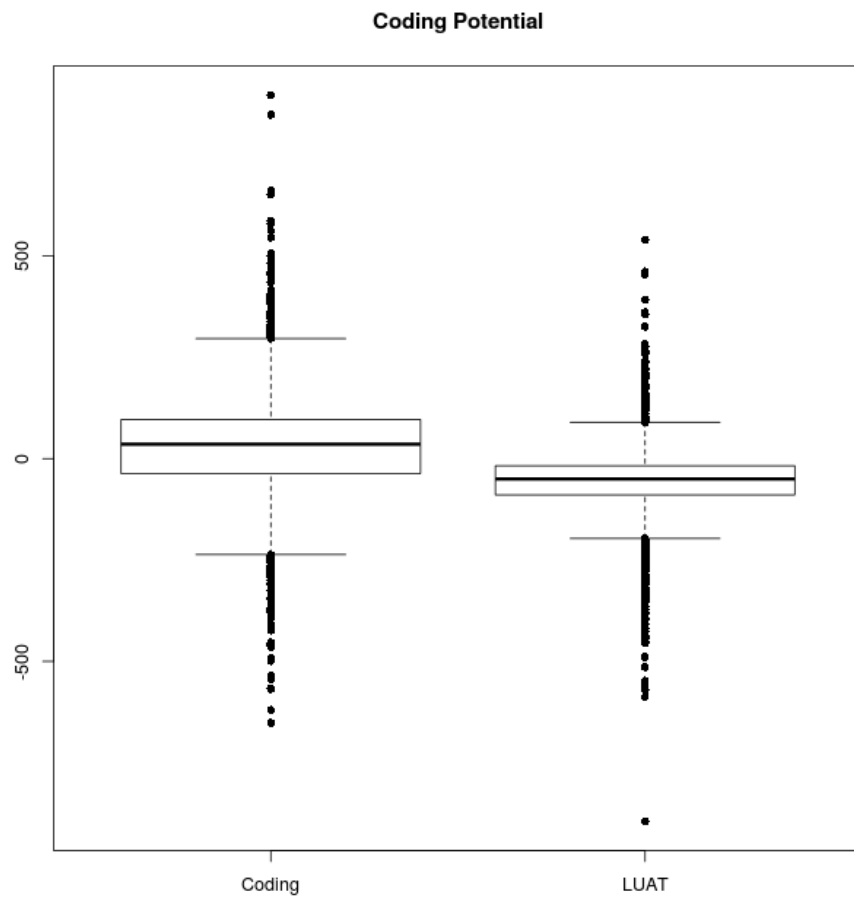

Supplement: Additional file 4: Figure S2 — Assessment of coding potential. PhyloCSF assessement of LUAT coding potential. The Galaxy web server (https://main.g2.bx.psu.edu/) was used to extract MAF blocks from 46-way multiZ alignments using cufflink transcript coordinates as input. Corresponding genomic sequences for human (hg19), Mus musculus (mm9), Rattus norvegicus (Rn4), Bos taurus (bosTau4) and Canis familiaris (canFam2) were retrieved for each block. Blocks shorter than 50 bp (95% of mouse exons) or missing one of the selected species were discarded. In order to create a positive control list, a set of blocks with same length distribution was randomly selected in exons from coding transcripts. The PhyloCSF program was used to assess coding potential of both sets. The resulting log-likelihood ratios are reported in units of decibans. [file 1471-2164-14-914-S4.pdf]

Expression levels of chromatin-, nucleoplasmic-, and cytoplasmic-associated transcripts

**A**

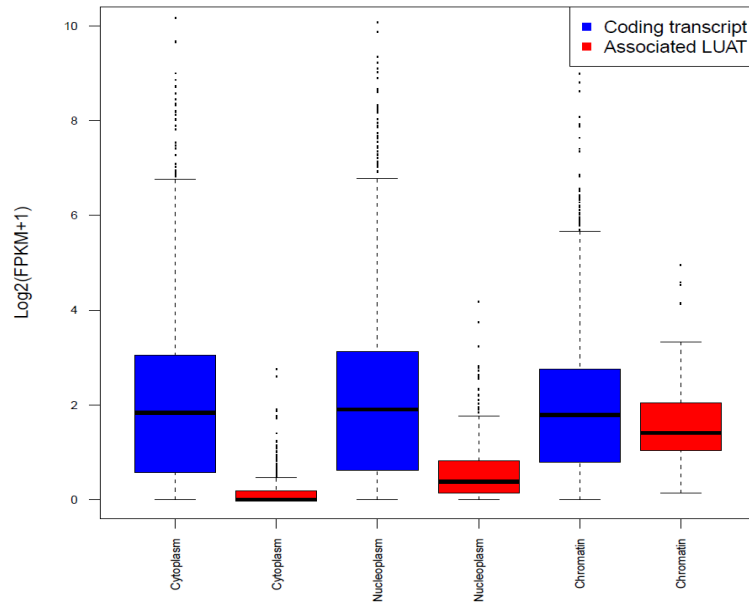

**B**

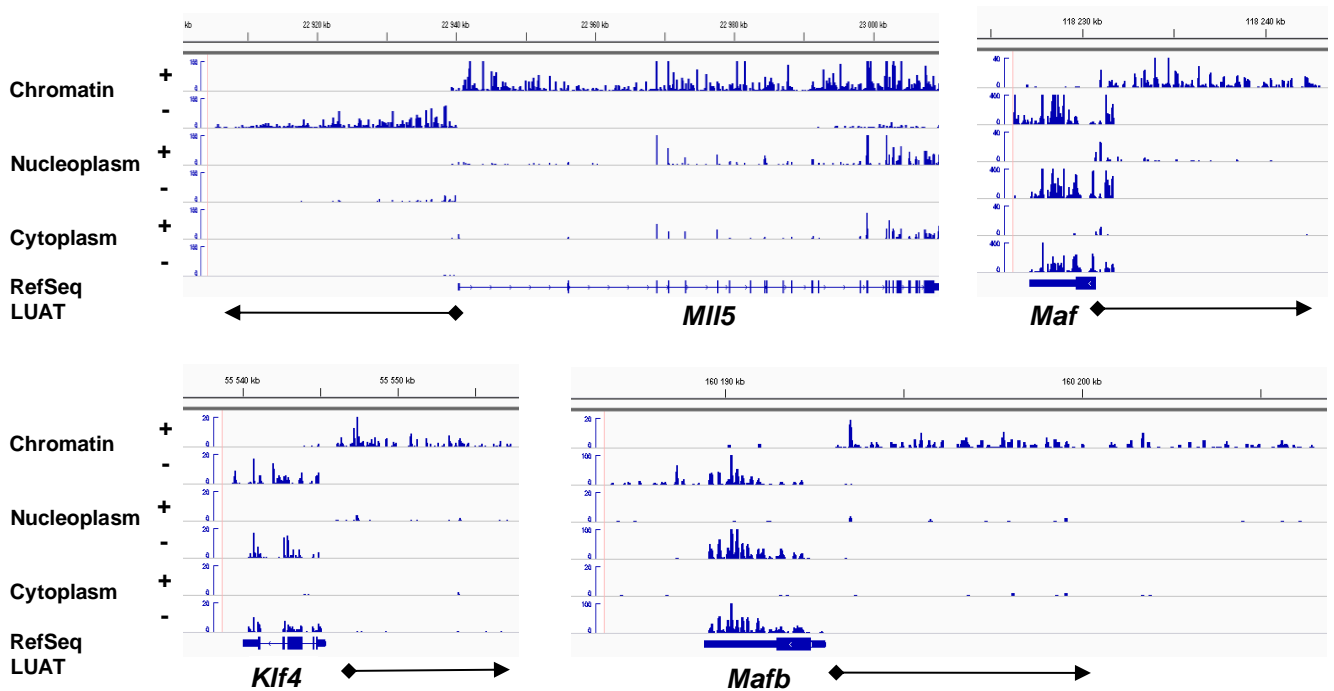

Supplement: Additional file 5: Figure S3 — LUATs are mainly found in the chromatin-associated fraction. To gain insight into the cellular localization of LUAT transcripts we analyzed recently published RNA-Seq data obtained from fractionated chromatin-associated, nucleoplasmic, and cytoplasmic transcripts from mouse macrophages (Bhatt et al. [27]; GEO serie: GSE32916). We performed assembly of divergent transcripts observed in these fractions (see “Identification of LUATs” in Methods section). A) boxplot displaying expression level as log2(FPKM + 1) for coding genes (blue) and LUATs (red) in the three different subcellular fractions. B) Representative examples of RNA-seq profiles from the three different subcellular fractions. Signal is provided for both plus and minus strands. The arrow highlights the presence of a LUAT. [file 1471-2164-14-914-S5.pdf]

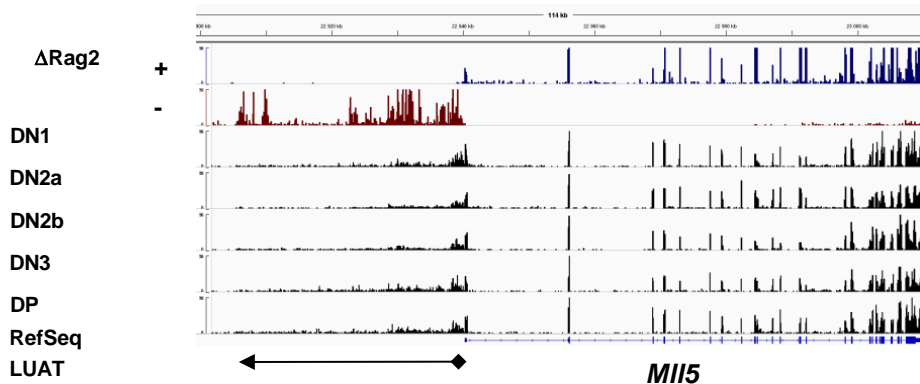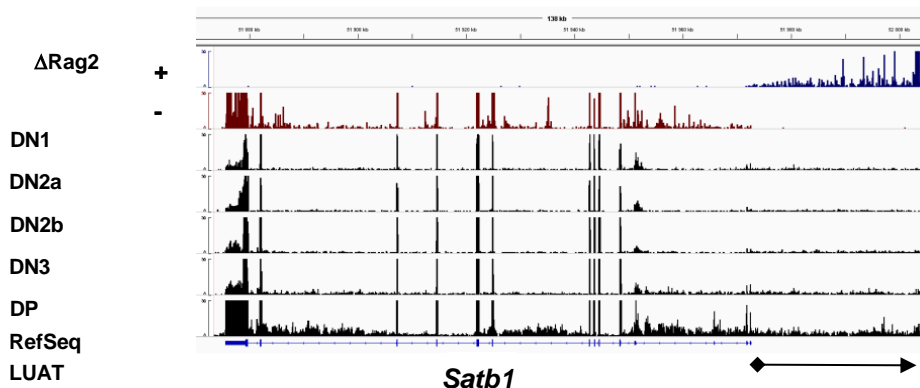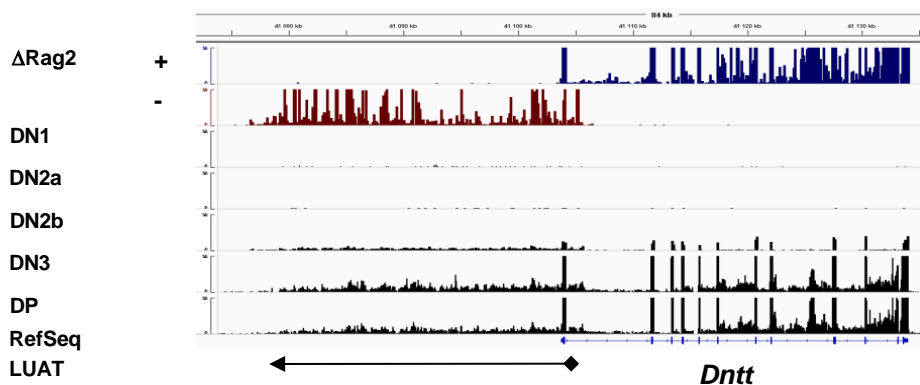

Supplement: Additional file 9: Figure S4 — Dynamic regulation of LUAT and their associated genes through early T-cell differentiation. In order to define expression profiles of LUAT and associated coding-genes through discrete stages of thymocyte development we retrieved unstranded PolyA RNA-seq from GEO web site (GSE31234, Zhang at al. 2012). Unstranded RNA-seq signal is shown for DN1, DN2a, DN2b, DN3 and DP (black track). Signal obtained from ΔRag DN thymocytes (SOLiD platform, this study) is also shown to highlight the expected signals from the plus (blue) and minus (red) strands. The arrow highlights the presence of a LUAT. [file 1471-2164-14-914-S9.pdf]

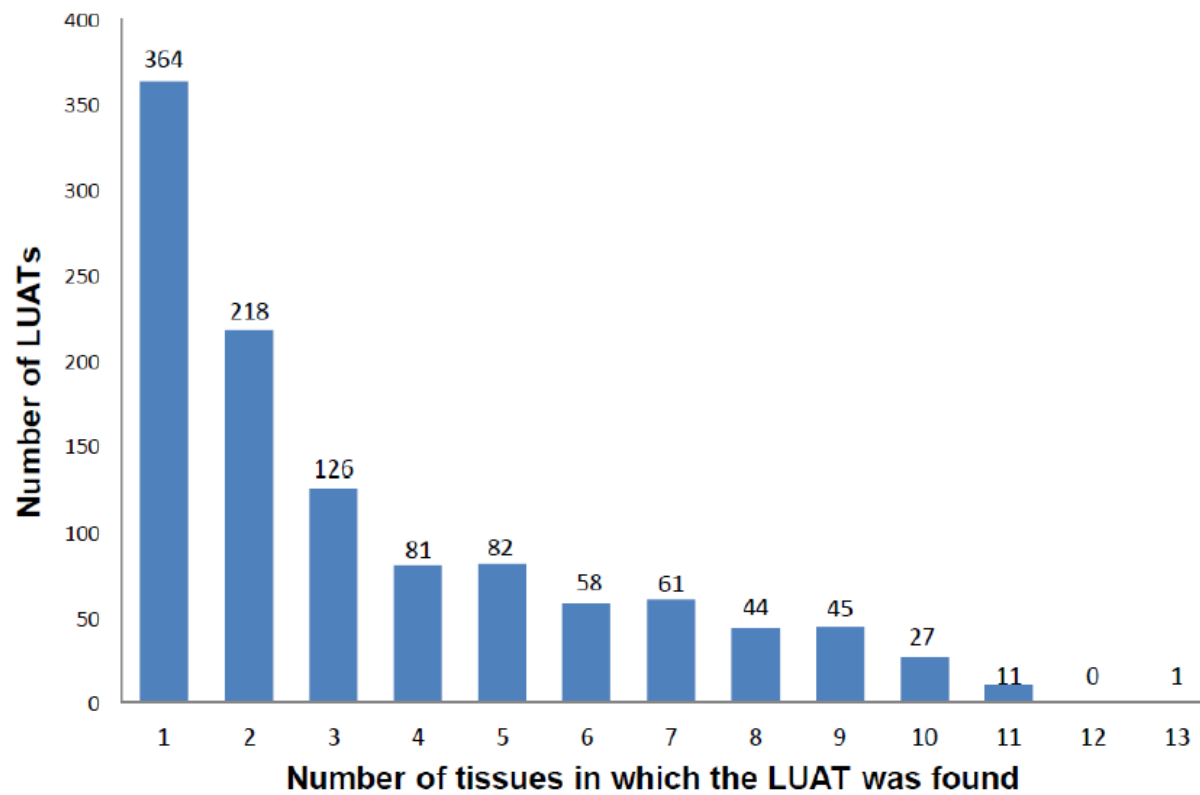

**Additional file 10 Figure S5**

Supplement: Additional file 10: Figure S5 — Tissue-specificity of LUATs. The histogram shows the number of tissues in which a given LUAT was found in the multi-tissue analysis. [file 1471-2164-14-914-S10.pdf]

**A**

## 5' splicing site motif distribution

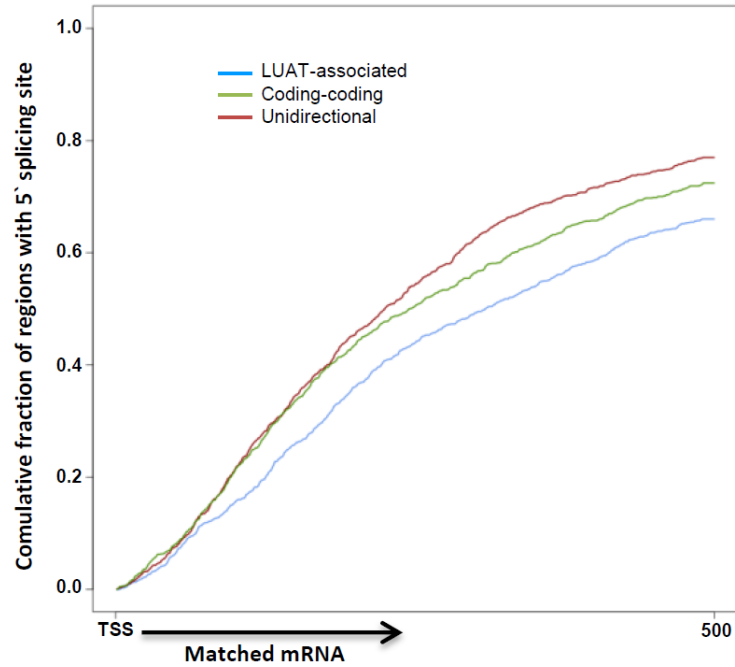**B**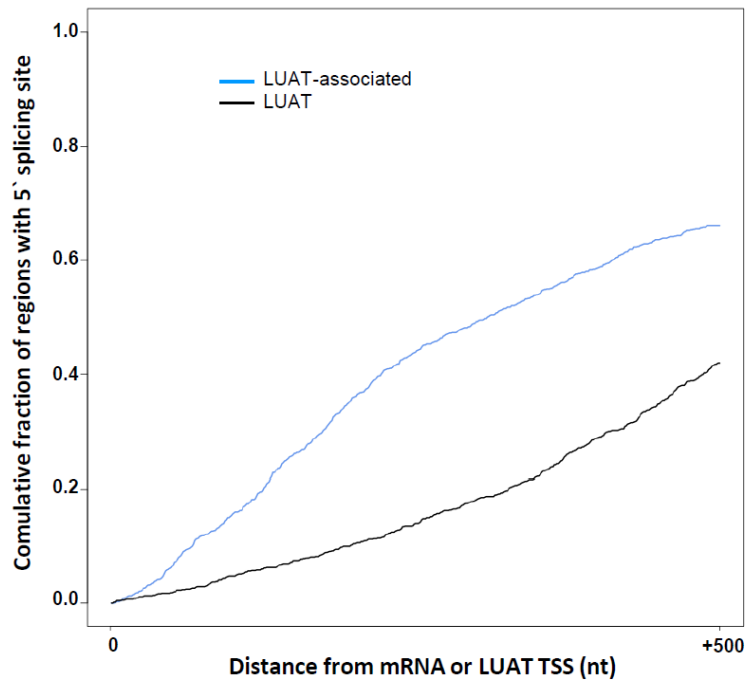

**Additional file 11 Figure S6**

Supplement: Additional file 11: Figure S6 — 5′ splice site distribution. Analysis for 5′ splice site motifs (Jaspar database; ID SD0001.1) in the 500 nt regions downstream of TSS for the three group of genes used for the multi-tissue analyses (A) or LUATs (B). The y axis shows the cumulative fraction of regions having at least one predicted site after traversal of a given number of nucleotides, as indicated on the x axis. [file 1471-2164-14-914-S11.pdf]

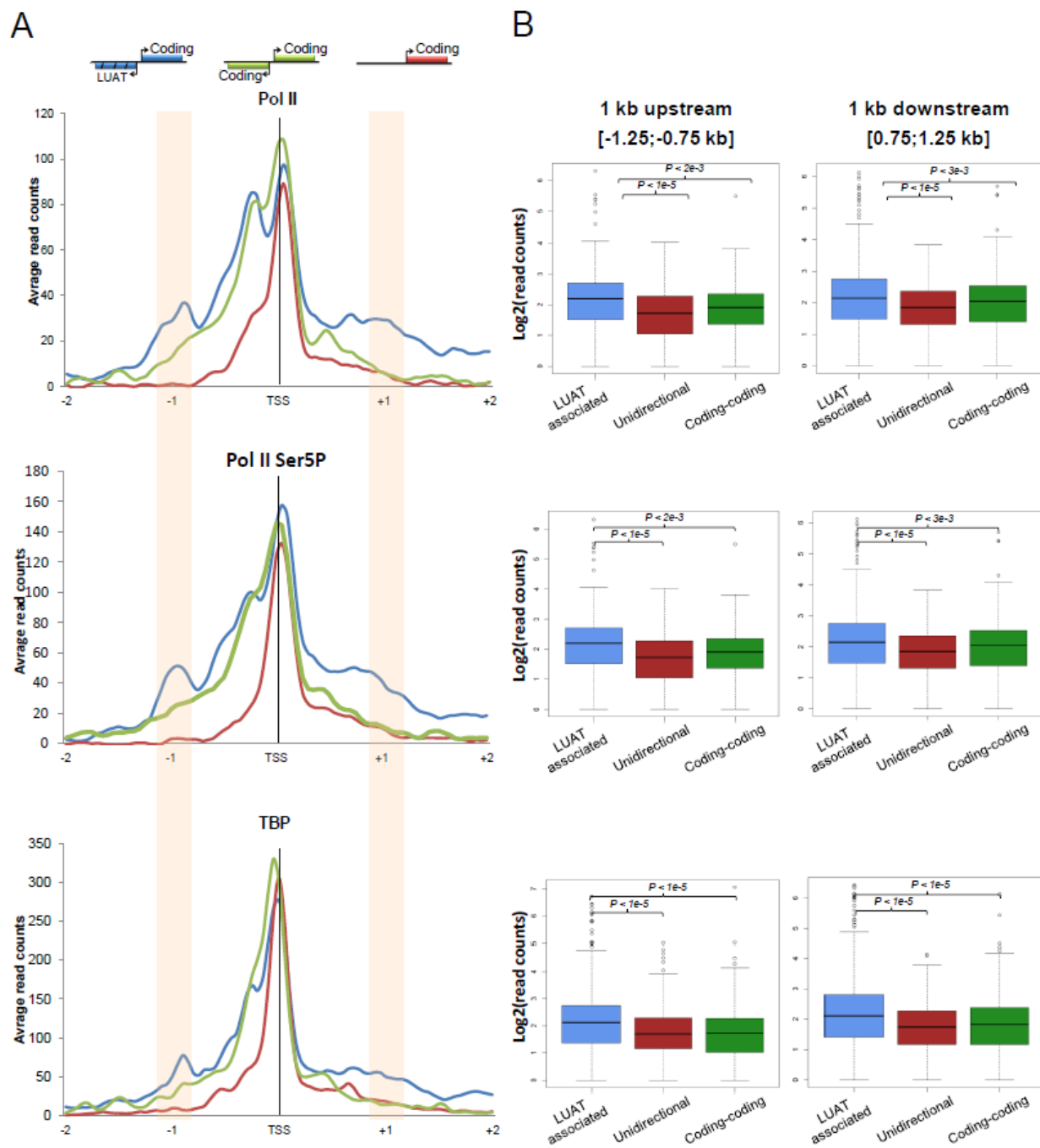

**Figure S7**

Supplement: Additional file 12: Figure S7 — Detailed view of TSS-centered ChIP-seq profiles for Pol II and TBP in DP thymocytes. Legends are as in Figure 6. The highlighted regions in pink correspond to the 500 nt regions analyzed in B. B) Number of reads within the indicated regions for the corresponding ChIP-seq experiments shown in A. The p-values of the Wilcoxon test are shown. [file 1471-2164-14-914-S12.pdf]

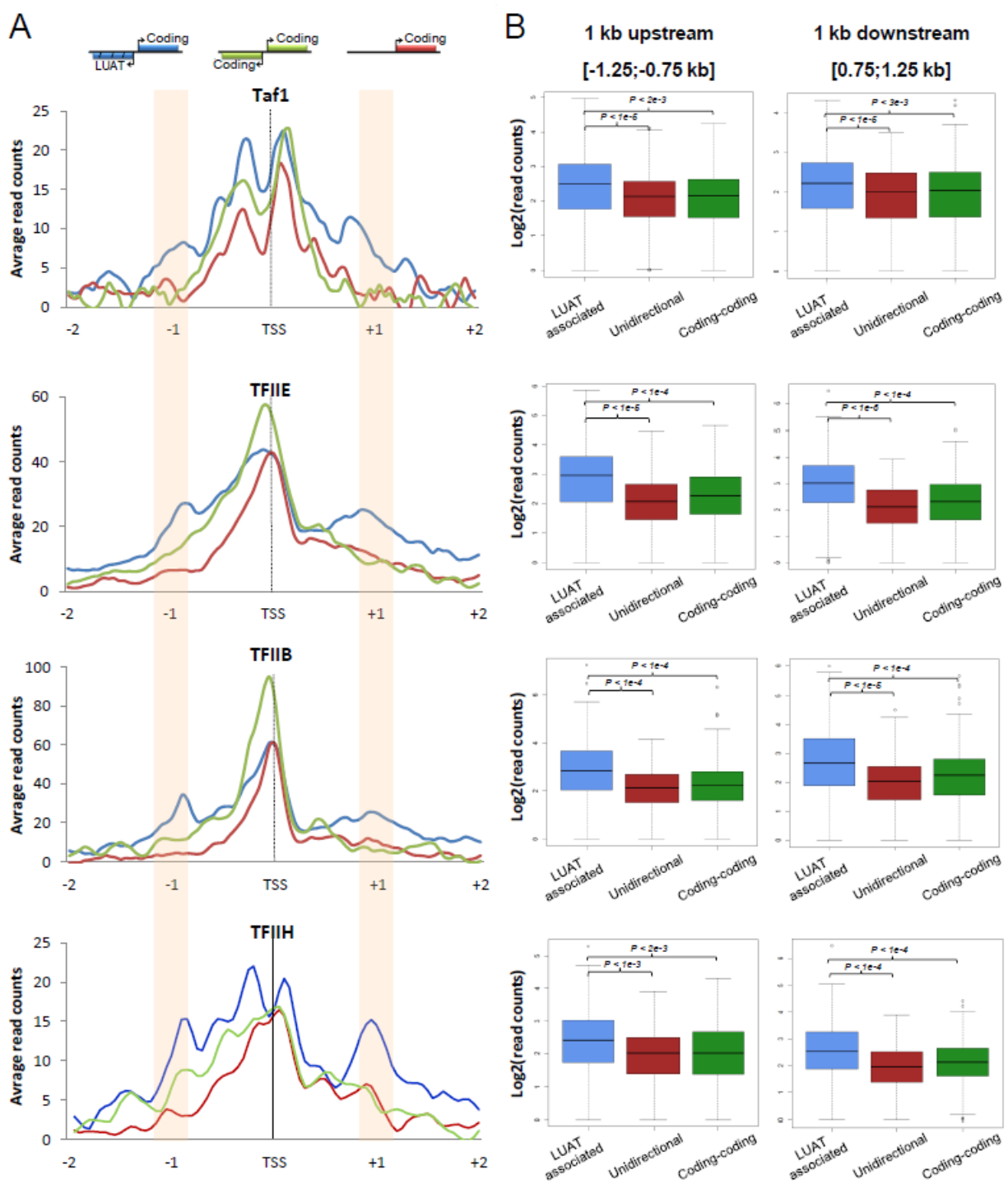

**Figure S8**

Supplement: Additional file 13: Figure S8 — Detailed view of TSS-centered ChIP-seq profiles for the indicated general transcription factors in DP thymocytes. Legends are as in Figure 6. The highlighted regions in pink correspond to the 500 nt regions analyzed in B. B) Number of reads within the indicated regions for the corresponding ChIP-seq experiments shown in A. The p-values of the Wilcoxon test are shown. [file 1471-2164-14-914-S13.pdf]
