# Supplementary material for: Divergent transcription is associated with promoters of transcriptional regulators
Source: BMC Genomics. 2013 Dec 23;14:914. doi: 10.1186/1471-2164-14-914 (PMC3882496; doi:10.1186/1471-2164-14-914)
Supplement: Additional file 14: Table S6 — Primers used for the RT-qPCR assays. [file 1471-2164-14-914-S14.doc]

**Table S6:** Primers used for the RT-qPCR assays

| **Gene** | **Location** | **Primers** | **Sequence** | **Category** |
| --- | --- | --- | --- | --- |
| Satb1 | Intron | F | GCTGTCTCCACTCAAAGCAG | Luat |
|  |  | R | TGTCCCCAAAAACTCATACATA | Luat |
|  | Exon | F | AAAGGGTGTTGGCGTTTTTA | Luat |
|  |  | R | GCTTGGAGGTGGATGTGG | Luat |
| Lef1 | Intron | F | GACTGAGGTTGGTTCCTGGT | Luat |
|  |  | R | GCCTGCGTGTATGTCTGTGT | Luat |
|  | Exon | F | TGACCCCAAGGAACACTGAC | Luat |
|  |  | R | AGACGACAGAAGGAAGAGGTG | Luat |
| Irf2 | Intron | F | TTACAGGGGACTTGCTGGAC | Luat |
|  |  | R | GGGGCTTAGAGTGTGGGTCT | Luat |
|  | Exon | F | CCACACTGGAGGAAGAGGAG | Luat |
|  |  | R | ATCTGGCTTGTTGGAGGTGA | Luat |
| Klf6 | Intron | F | AAACCCAGGAAAAGCGAAGT | Luat |
|  |  | R | GAAAAATGGAGAGGGGGAGA | Luat |
|  | Exon | F | GAAGGAGCAGAGGGACGAAT | Luat |
|  |  | R | AACTGGATAGGAAGCAAAATGG | Luat |
| MLL5 | Intron | F | ATTGAGAACTTTTGCCCATTTC | Luat |
|  |  | R | CATTGCTGCTACCCATAACC | Luat |
|  | Exon | F | TCTACAAGCACAACACCAGCA | Luat |
|  |  | R | ATGCCCAGAAACCAGAACAC | Luat |
| TCF7 | Intron | F | GCGGTCTCTCAGGTTTGCTA | Luat |
|  |  | R | AGGCTGGAACTCACCATCAG | Luat |
|  | Exon | F | GAGGTCCAGCCAGCAATAAC | Luat |
|  |  | R | TCAAGAGGTGGGGGATTAGA | Luat |
| Myb | Intron | F | GCCATTTCCTTGCTCTAAAA | Luat |
|  |  | R | CCTTTGACTGTTGGGTCTGAG | Luat |
|  | Exon | F | AGGAGCCATGTGTCGAAAGT | Luat |
|  |  | R | GCGTTCTCAGCTCGAACTCT | Luat |
| Tox | Intron | F | AACCATTTTGGGGGAGAATC | Luat |
|  |  | R | CGGGCATTTGTCTTCACATA | Luat |
|  | Exon | F | CAGGACCGTTTACCCCAGA | Luat |
|  |  | R | TCCCAGAAGGAAGTGTGGAC | Luat |
| Rorc | Intron | F | CGTGGTTCTTTTCTGGCTTC | Luat |
|  |  | R | GCTGTTGCCCTCTGCTTC | Luat |
|  | Exon | F | AGGACAACAGCAGCAAGTGA | Luat |
|  |  | R | TATCCCATCTACCCCACAGC | Luat |
| Ikzf1 | Intron | F | CCTCTCCTCAGTGGCTGTG | Luat |
|  |  | R | CTCTCTCCTCCCCCAGGTAA | Luat |
|  | Exon | F | AGCATCGTTACCACCTGAGC | Luat |
|  |  | R | GCAAATCCACTCCCAACATT | Luat |
| Asxl1 | Intron | F | ATGGAGGGGGAGATAAAGGA | Luat |
|  |  | R | TCTACCCGACTTCTCCCAAG | Luat |
|  | Exon | F | AGAGACAGCAGCAGTGGTGA | Luat |
|  |  | R | GACAAGGCGGCAGTAGTTGT | Luat |
| Fads1 | Intron | F | TTCAGCGGAGTCTTTCCAGT | Unidirectional |
|  |  | R | TCCTCCCTCCTCCCTACTGT | Unidirectional |
|  | Exon | F | TCTTCCCTCCCACTGAACAC | Unidirectional |
|  |  | R | CTCTGGTGGAAGCCTCAGTC | Unidirectional |
| Taok | Intron | F | AGTGCTAGGCAGGGTGAGAA | Unidirectional |
|  |  | R | ACCTCTGCATGTGTGTCTGC | Unidirectional |
|  | Exon | F | AGAAGGAACGCAGTGAGAGG | Unidirectional |
|  |  | R | AAATTCCCAAAGCCCATTCT | Unidirectional |
| Cdk5rap2 | Intron | F | GGGTCAAAGAAGCCAAACAA | Unidirectional |
|  |  | R | TGCCAAGCACAGTTCAAAAG | Unidirectional |
|  | Exon | F | CTTTCTCCTGGTGCTTGCTC | Unidirectional |
|  |  | R | TGAAGGCAGAGAACACGAAA | Unidirectional |
| Prkci | Intron | F | TGTGTTGGCGATTGGTTCTA | Unidirectional |
|  |  | R | GCCGTGTTGGCATCTTTATT | Unidirectional |
|  | Exon | F | GAAGCCTGGAGTTGGAACAG | Unidirectional |
|  |  | R | GACAGTTGCTGACTGGTGGA | Unidirectional |
| Ndufa5 | Intron | F | CCAGGACAGCGGAACACT | Coding-coding |
|  |  | R | ACCGTGATGCTGCGAATG | Coding-coding |
|  | Exon | F | CCATCCACCATCTGACACTG | Coding-coding |
|  |  | R | TCTGGCAAGGAAAATGTTGA | Coding-coding |
| Psmc5 | Intron | F | TCGAAGGTAGGCGGTAATTG | Coding-coding |
|  |  | R | GCGAGGTCTGTGTGTTTGAA | Coding-coding |
|  | Exon | F | TCATGCAAAAGGATAGTGAGAAA | Coding-coding |
|  |  | R | CACACAGACCTTTATTGGTTGG | Coding-coding |
| Phf4a | Intron | F | AGCAATCTTCCAGCCTTCTG | Coding-coding |
|  |  | R | TGACATGGCCTTTGGTGTAA | Coding-coding |
|  | Exon | F | GTCACAGGACAACCCCATCA | Coding-coding |
|  |  | R | TCAACTCAAAAGCCTCAAAACA | Coding-coding |
| Tax1BP3 | Intron | F | CTTTGCTGCCTGACTTCTCC | Coding-coding |
|  |  | R | GGCTTACTCCCACACACACC | Coding-coding |
|  | Exon | F | GTTCTGGCTTCTGCTGTGTG | Coding-coding |
|  |  | R | TGGGAGGTTGAGGGATAAGA | Coding-coding |
| Brap | Intron | F | GGCACTAGGCACAAACAGGT | Coding-coding |
|  |  | R | CCGATCCACACATCTAAGCA | Coding-coding |
|  | Exon | F | TGCGCGATGTCATGTTCTAC | Coding-coding |
|  |  | R | ATGTTGATCTGGCCTTCCTG | Coding-coding |
